# Supraclavicular Foramina in Dry Clavicles: An Observational Study and Meta-Analysis of Prevalence and Laterality

**DOI:** 10.3390/jfmk11030289

**Published:** 2026-07-24

**Authors:** Vasileios Papadopoulos, Vagia Tourtouri, Andromachi Navrozidou, Athanasios Markou, Petros Siakavellas, Aliki Fiska

**Affiliations:** Laboratory of Anatomy, Department of Medicine, Democritus University of Thrace, Dragana, 68100 Alexandroupolis, Greece; vagitour@med.duth.gr (V.T.); andrnavr@med.duth.gr (A.N.); athamark2@med.duth.gr (A.M.); petrsiak@med.duth.gr (P.S.); afiska@med.duth.gr (A.F.)

**Keywords:** supraclavicular nerve, transclavicular canal, anatomical variation, clavicle morphology, intraosseous nerve canal, prevalence, laterality, meta-analysis

## Abstract

**Background:** The supraclavicular foramen (SCF) is a rare anatomical variant formed when a branch of the supraclavicular nerve traverses the clavicle through a complete transclavicular canal. Published studies differ substantially in study material, denominators, and reporting of side-specific and bilateral data, so bilateral anatomy is often synthesized as if pairing were irrelevant. **Objectives**: This study aimed to describe SCF in a Greek osteological sample and to apply a meta-analytic framework distinguishing per-clavicle prevalence, marginal laterality, paired laterality, and separately observed bilateral prevalence. **Methods**: We examined 115 dry clavicles of Greek origin for SCF frequency, size, and topography. Measurements were obtained independently by four observers using stainless-steel wires and digital calipers, with inter-rater agreement assessed by intraclass correlation coefficients. A systematic review and meta-analysis were then conducted according to Preferred Reporting Items for Systematic Reviews and Meta-Analyses (PRISMA) 2020 guidelines. PubMed, Scopus, and SciELO were searched from inception to 11 April 2026, supplemented by Google Scholar and backward and forward citation tracking, without language restrictions. Quantitative synthesis included pooled per-clavicle prevalence, conventional marginal left-versus-right comparison, and feasibility-based paired laterality analysis under explicit dependence assumptions when bilateral information was incomplete. Observed bilateral prevalence was analyzed separately using only studies with directly reported body-level bilateral counts. Risk of bias in the included studies was assessed using the Anatomical Quality Assessment tool, while the review itself was appraised using the Critical Appraisal Tool for Anatomical Meta-Analysis. **Results**: Complete transclavicular canals, identified by two external openings connected by a complete transosseous passage, were present in 3/115 clavicles (2.6%), with a mean aggregate transverse wire span of 1.5 ± 0.8 mm and a mean relative acromial position of 0.44 ± 0.01. Inter-rater reliability was excellent. Pooled per-clavicle prevalence was 2.2%, with 95% confidence intervals (CIs) 1.4–3.0% and I^2^ = 71%. Conventional marginal laterality supported left-sided predominance, with an odds ratio of 1.76 (95% CI: 1.16–2.66; I^2^ = 0%), while feasibility-based paired laterality yielded a stronger effect (paired OR = 2.26, 95% CI: 1.30–3.94). The main observed bilateral-prevalence model yielded 0.95% (95% CI: 0.33–2.70%). The Doi plot and Luis Furuya-Kanamori index indicated minor asymmetry. **Conclusions**: Complete transclavicular canals occurred in 2.6% of the examined clavicles and occupied a reproducible mid-to-lateral position. The meta-analysis confirmed that the variant is uncommon, supported left-sided predominance, and showed that directly observed bilateral occurrence is rare. Separating clavicle-level prevalence, marginal laterality, feasibility-based paired laterality, and body-level bilateral prevalence provided a more appropriate synthesis of incompletely paired evidence.

## 1. Introduction

The supraclavicular foramen is an uncommon clavicular variant, with most published series reporting frequencies below a few percent, although higher values have been described in selected populations. Reported prevalence varies according to population, study material, and detection modality, and the contribution of biological variation cannot always be separated from differences in ascertainment. The occurrence of the variant in immature individuals and its clustering in some relatively isolated populations support a developmental and partly genetic basis, whereas side-specific persistence may also be influenced by postnatal mechanical remodeling [1].

**Figure 1:**
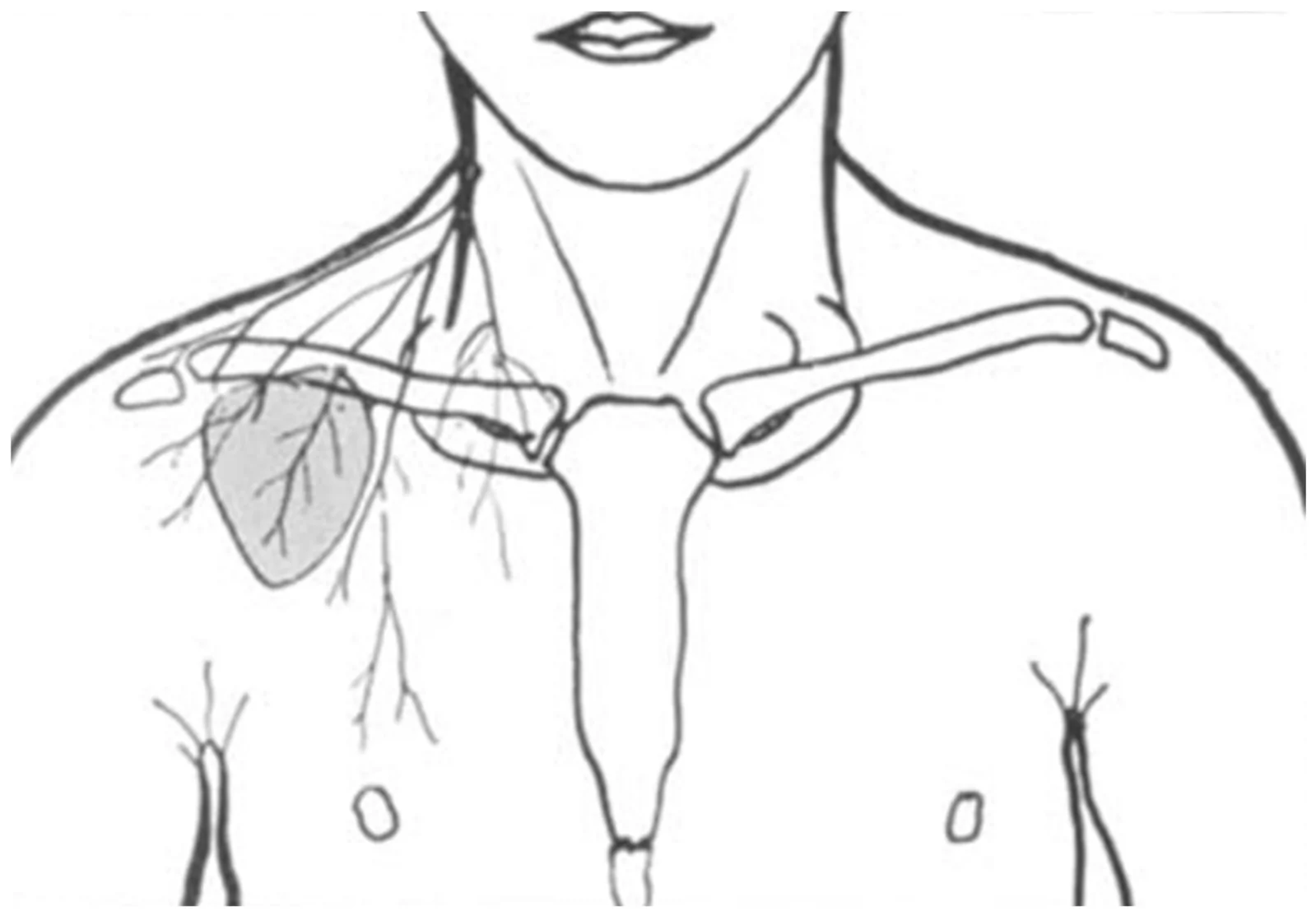
Schematic overview of the supraclavicular nerve branches and the cutaneous territory of the intermediate branch (adjusted from Skarby).

The supraclavicular nerves consist of three cutaneous branches—medial, intermediate, and lateral—arising from the C3 and C4 spinal nerves of the cervical plexus [2,3]. After emerging beneath the posterior border of the sternocleidomastoid muscle, they descend superficially to supply sensation to the skin over the clavicle, shoulder, and upper thoracic region (Figure 1). Because of their superficial course, they are vulnerable to injury during clavicular and shoulder surgery or following trauma to the neck and upper thorax [4].

Marked anatomical variability has been described in the origin, course, branching pattern, and clavicular relationships of the supraclavicular nerves [2,3]. These variations carry important clinical implications, influencing surgical exposure, anesthetic blocks, and postoperative sensory outcomes. A thorough understanding of these patterns is therefore essential to reduce iatrogenic complications and improve regional anesthesia success.

Normally, the clavicle contains a single nutrient canal on its inferior surface, presumably transmitting the main nutrient artery and terminating blindly without an external opening [5]. In rare cases, however, a complete transclavicular canal may be present. This osseous passage traverses the full thickness of the clavicle and typically transmits a branch of the supraclavicular nerve—most commonly the intermediate branch—giving rise to two external openings, referred to here as supraclavicular foramina (SCF) [1]. Thus, transclavicular canal denotes the complete intraosseous passage, whereas supraclavicular foramen denotes either external opening of that passage. Such a course contrasts with the typical pattern, in which the nerves cross superficially over the clavicle.

The formation of these transclavicular canals is thought to result from entrapment of a nerve branch during clavicular ossification. Because these canals do not have the structure of the perforating canals of Volkmann, the supraclavicular nerves are unlikely to have pierced fully formed bone and were more probably enclosed during later bone formation [6]. They usually occur within the midshaft of the bone and only rarely near the acromial end. Their depth and configuration may vary, and bony bridges can occasionally form, creating a roof over the nerve’s trajectory. As a result, an entrapped nerve may be susceptible to injury in cases of clavicular fractures [7].

Although uncommon, supraclavicular nerve entrapment syndrome should be considered among the causes of anterior shoulder girdle pain. Entrapment may occur due to anomalous bone structures, fibrous bands, or muscular variations [8]. When the nerve passes through an intraosseous canal, symptoms such as pain or numbness most frequently involve the intermediate branch. Surgical decompression may provide symptom relief. Thus, despite its rarity, clinicians should include intraclavicular supraclavicular nerve entrapment in the differential diagnosis of shoulder pain [9,10].

Because the transclavicular canal is the term most often used in the anatomical and radiological literature, it is retained here for the complete osseous passage, whereas supraclavicular foramen is used for the external opening and as the preferred general term for the variant.

Despite nearly two centuries of anatomical descriptions, the literature remains limited by inconsistent terminology, heterogeneous dry-bone, cadaveric, and imaging materials, sparse morphometric reporting, and incomplete documentation of donor-level pairing and bilateral occurrence. Previous syntheses have generally emphasized clavicle-level prevalence or marginal side counts, whereas directly observed bilateral prevalence and reconstructed paired laterality address different anatomical questions. A synthesis that explicitly separates these estimands is therefore needed.

The present study aimed to characterize the prevalence, morphology, and topography of complete transclavicular canals in a Greek osteological sample and to synthesize the published evidence across four distinct but complementary estimands: per-clavicle prevalence, conventional marginal laterality, paired unilateral laterality, and directly observed bilateral prevalence. Per-clavicle prevalence estimates how often an individual clavicle bears a complete canal, whereas marginal laterality compares observed left- and right-sided frequencies without accounting for donor-level pairing. Paired laterality instead compares left-only with right-only occurrence at the individual level and, when bilateral counts are incompletely reported, requires explicit assumptions regarding within-individual dependence. Directly observed bilateral prevalence was therefore analyzed separately using only studies with an identifiable body-level denominator and reported bilateral cases. We expected SCF to be uncommon and explored whether its frequency varied according to side and study material; we also examined whether accounting for the paired structure of bilateral anatomy altered the laterality estimate relative to the conventional marginal comparison. By separating these estimands, the study also evaluates the broader methodological principle that bilateral anatomical data should be analyzed using models matched to their underlying unit of observation and dependence structure.

## 2. Materials and Methods

### 2.1. Present Observational Study

#### 2.1.1. Specimen Selection

This investigation assessed the frequency, size, and anatomical position of supraclavicular foramina in 115 dry human clavicles (60 right, 55 left) of Greek origin. At the time of data collection, the osteological collection contained 126 adult human clavicles. Of these, 11 were excluded because of fracture (*n* = 1), deformity (*n* = 1), erosion (*n* = 1), incomplete preservation (*n* = 7), or uncertain side (*n* = 1), leaving 115 eligible specimens. All eligible clavicles were included, and no additional sampling was performed. The skeletal material was archived at the Anatomy Laboratory, Department of Medicine, Democritus University of Thrace, Greece. Information on age and sex was unavailable. Because the material was derived solely from cadaveric collections, institutional ethics approval was not required.

The clavicles were stored as individual specimens and lacked donor-level identifiers permitting reliable left–right pairing. Consequently, the observational sample was analyzed at the clavicle level and was not treated as a paired bilateral sample.

#### 2.1.2. Measurements

Each clavicle was inspected under direct light for the presence of a complete transclavicular canal, identified by two external supraclavicular foramina corresponding to two external openings connected by a complete transosseous canal through the bone. The primary objectives were to determine largest-single-wire patency, aggregate transverse wire span, external opening dimensions, and canal position relative to the sternal and acromial ends of the clavicle.

A complete transclavicular canal was confirmed only when a flexible stainless-steel wire passed continuously between two external openings on the same clavicle. Internal canal patency was assessed using stainless-steel wires ranging from 0.2 to 1.2 mm in diameter (UA218893, Zhejiang, ZHEJIANG WANSHENG YUNHE STEEL CABLE Co., Ltd., Hangzhou, China), following previously described procedures [11]. For each canal, progressively larger wires were tested, and the largest individual wire that traversed the complete passage was recorded as the largest-single-wire patency. If a wire failed to pass, the next smaller available gauge was tested; thus, the recorded value represented the largest confirmed traversing diameter, with a maximum discretization error of 0.1 mm. Because the canal lumen was non-circular, equal-gauge wires were also aligned side by side along the widest accessible internal dimension and passed simultaneously through the canal until no additional wire could be accommodated. The aggregate transverse wire span was calculated as the number of simultaneously traversing wires multiplied by the diameter of each wire. This operational measure was interpreted as a lower-bound estimate of the wider internal lumen dimension rather than as a directly measured anatomical diameter. Patency below the smallest available wire diameter of 0.2 mm could not be assessed.

The dimensions of both external openings of each complete canal were additionally measured with a Mitutoyo digimatic caliper (Mitutoyo Co., Kawasaki, Japan; accuracy 0.01 mm). Canal length was defined as the inter-opening distance. Canal location was summarized by the midpoint of the two openings, and foraminal position was expressed relative to the acromial and sternal ends of the clavicle and to total clavicular length. An approximate projected canal obliquity (φ) was calculated from the longitudinal displacement of the two openings along the clavicular axis. Specifically, if L denotes the inter-opening distance (canal length) and Δx denotes the absolute difference between the sternal distances of the two external openings, then projected obliquity was defined by sin(φ)=Δx/L, and therefore, φ=arcsin(Δx/L). This formulation expresses the angle of the canal relative to a purely anteroposterior course in its projected clavicular plane and was used as a descriptive geometric estimate rather than as a true three-dimensional angular measurement. Representative caliper-based measurement of SCF dimensions is shown in Supplementary Figure S1.

All morphometric measurements were obtained independently by four observers (V.P., V.T., A.N., and A.M.). Final specimen-level values were calculated as the arithmetic mean of the four observer measurements. Inter-rater reliability was assessed using a two-way mixed-effects, absolute-agreement, average-measures intraclass correlation coefficient [ICC(A,4)] with 95% confidence intervals.

#### 2.1.3. Statistical Analysis of the Observational Sample

The analytical strategy for continuous morphometric variables was contingent on the number of complete transclavicular canals identified. Formal assessment of distributional form and inferential comparisons were undertaken only when the number of observations was sufficient to make such analyses meaningful. When the number was insufficient, measurements were summarized descriptively using individual values and mean ± standard deviation, solely to facilitate comparison with previous morphometric reports. Mean ± standard deviation was used as a descriptive summary and was not intended to imply normality. For the complete set of three observed canals, aggregate transverse wire span was summarized using the population standard deviation.

Continuous variables are reported as mean ± standard deviation (SD). Categorical variables are reported as counts and percentages. Owing to the rarity of the variant, the observational component was interpreted primarily descriptively. Statistical analyses were performed using SPSS version 26.0 (IBM Corp., Armonk, NY, USA).

### 2.2. Systematic Review and Meta-Analysis

#### 2.2.1. Search Strategy and Study Selection

The systematic review was conducted in accordance with the Preferred Reporting Items for Systematic Reviews and Meta-Analyses (PRISMA 2020) guidelines [12]. Two reviewers independently searched PubMed, Scopus, and SciELO from database inception to 11 April 2026, without language restrictions. Only studies of human clavicles, human cadaveric specimens, or human imaging datasets were eligible. Animal and non-human primate studies were excluded. The PubMed query was “(supraclavicular foramen) OR (transclavicular canal)”, and the Scopus query was “(supraclavicular AND foramen) OR (transclavicular AND canal)”. In SciELO, the terms “supraclavicular foramen” and “transclavicular canal” were searched separately. Google Scholar was searched as a supplementary source on 11 April 2026 using the same terms, and the first 200 results were screened. Backward and forward citation tracking was also performed for all included reports. The supplementary Google Scholar search identified one additional eligible publication. No separate systematic search of theses or conference proceedings was performed.

After duplicate removal and screening by title and abstract, eligible full-text articles were assessed for inclusion. Studies describing the frequency, morphology, or topography of supraclavicular foramina or complete transclavicular canals in dry clavicles, cadaveric material, or radiological investigations (plain radiography or CT) were considered eligible.

Disagreements during screening or eligibility assessment were resolved through discussion. If consensus could not be reached, a third reviewer adjudicated.

#### 2.2.2. Data Extraction and Study Classification

From each included study, two reviewers independently extracted the following information: first author, year, country, study material (dry bones, cadaveric tissue, radiography, or CT), total number of clavicles, right/left distribution where available, number of clavicles with transclavicular canals, and body-level bilateral information where explicitly reported. Morphometric variables, such as foraminal diameter and location relative to clavicular ends, were also recorded when available. For historical studies reporting only case-wise measurements or range summaries, morphometric data were additionally abstracted in a form suitable for descriptive reconstruction.

A standardized extraction form was piloted before formal data collection. Two reviewers independently extracted study characteristics, denominators, event counts, side-specific data, body-level bilateral information, and morphometric variables. Extracted values were compared and reconciled by discussion, with third-reviewer adjudication when required. Data were taken preferentially from numerical tables and main text; values were calculated directly from reported numerators and denominators when possible. Historical tables and figures were transcribed independently and cross-checked. Missing information was coded as not reported and was not inferred, except for the prespecified feasibility-based and range-based reconstructions described below. Corresponding authors were not contacted for unavailable information.

Disagreements during data extraction were resolved through discussion. If consensus could not be reached, a third reviewer adjudicated.

Because studies differed in whether they reported clavicle-level, side-specific, or body-level data, the evidence base was classified according to the inferential information each study could support. Specifically, studies were categorized according to their contribution to (i) per-clavicle prevalence, (ii) conventional marginal laterality, (iii) feasibility-based paired laterality, and (iv) main observed bilateral prevalence.

As shown in Table 1, studies with side-specific right/left counts were eligible for laterality analysis, whereas known body-level denominators and observed bilateral counts were incorporated only when explicitly reported. A list of full-text studies excluded after eligibility assessment, with reasons for exclusion, is provided in Supplementary Table S1.

#### 2.2.3. Quality Appraisal and Risk of Bias Assessment

Methodological quality and risk of bias were assessed independently by two reviewers using the Anatomical Quality Assessment (AQUA) tool [13]. Each AQUA domain was rated as low, unclear, or high risk of bias, whereas an overall methodological quality rating was assigned separately for summary appraisal as reported elsewhere [14]. The overall robustness of the present review was additionally evaluated using the Critical Appraisal Tool for Anatomical Meta-Analysis (CATAM) [15]. In light of recent psychometric evidence indicating that CATAM total-score reliability is inadequate for a single rater but acceptable when ratings are aggregated across four independent raters, CATAM was applied independently by four reviewers (V.P., V.T., A.N., and A.M.) [16]. Each reviewer scored the manuscript separately, and domain scores were then compared across raters. Because CATAM was applied to the present manuscript as a single object of appraisal, inter-rater reproducibility was summarized descriptively by exact agreement rather than by an intraclass correlation coefficient.

Risk of bias was mitigated through duplicate independent screening and data extraction, prespecified eligibility criteria, explicit separation of observed and reconstructed outcomes, study-level AQUA appraisal, four-rater CATAM appraisal of the present review, sensitivity analyses for transformation choice and dependence assumptions, and complementary small-study-effect diagnostics.

#### 2.2.4. Descriptive Synthesis of Morphometric Data

Morphometric data on transclavicular canals were synthesized separately from the main meta-analytic models. Because the available studies reported sparse canal-level measurements with heterogeneous definitions and incomplete person-level linkage, no formal random-effects meta-analysis was performed for canal location or size. Instead, canal-count-weighted descriptive data were summarized in an appropriate table.

When directly comparable mean ± SD values were available, these were extracted as reported. For historical studies that provided only ranges or grouped summaries, approximate mean and SD values were reconstructed under an assumption of approximate normality. In Santos (1927) [17], which described 30 complete canals, the sample mean of a variable reported only by its minimum and maximum was approximated by the mid-range, and the SD was estimated using the range-based method of Wan et al.:

$$SD\approx \frac{max-min}{2\Phi^{-1}\left(\frac{n\;-\;0.375}{n\;+\;0.25}\right)}$$

where n is the sample size and $\Phi^{-1}$ is the standard normal quantile function [17,18]. This approach was applied to Santos-derived canal length and acromial-distance summaries.

Because Santos reported distances from the acromial end to the anterior and posterior canal orifices rather than a standardized canal-margin measure, the distance from the acromial end to the anterior (closest) orifice was used as the best available approximation of lateral distance. The lateral distance/total clavicle length ratio was then derived from Santos’ grouped clavicle-length and anterior-orifice-distance summaries. For the ratio, SD was approximated heuristically from the reconstructed range under the same approximate-normality framework. Santos’ reported orifice diameters were not entered into the modern canal-width column because they are not directly comparable with later studies reporting maximum canal diameter [17].

Potential within-individual dependence of canal-level morphometric observations was examined in a sensitivity analysis. In Santos (1927), three bilateral skeletons were reported, implying six potentially correlated canal observations among the 30 complete canals [17]. Those six observations were downweighted using an effective-sample-size correction

$$n_{eff}=n_{unpaired}+\frac{n_{paired}}{1+r}$$

with r=0.75, consistent with the midpoint attenuation approach proposed for repeated-measures settings when the true correlation is unobserved [19]. The remaining 24 canals retained full weight. Because person-level linkage was unavailable for the other morphometric datasets, no study-specific correction could be justified for them; morphometric summaries were therefore interpreted descriptively, and the pairing-adjusted calculation was retained as a sensitivity analysis only.

#### 2.2.5. Statistical Synthesis of Prevalence, Marginal Laterality, Paired Laterality, and Bilateral Prevalence

Because SCF has been studied in dry clavicles, cadaveric series, radiographs, and computed tomography datasets with variable or incompletely reported pairing, quantitative synthesis was structured on distinct inferential scales. The first scale was per-clavicle prevalence, treating the clavicle as the anatomical unit of analysis. The second was conventional marginal laterality, comparing the observed frequency of SCF in left versus right clavicles without modeling within-individual dependence. The third was paired individual-level laterality, targeting unilateral left-only versus right-only occurrence under a feasibility-based reconstruction of the unobserved joint left-right distribution when studies reported side-specific marginals but not complete bilateral counts. In parallel, observed bilateral prevalence was analyzed separately using only studies with directly reported body-level bilateral data. This separation was prespecified to distinguish what was directly observed from what was reconstructed under explicit assumptions.

All quantitative syntheses were conducted under a random-effects framework to account for between-study heterogeneity in population characteristics, material type (dry bones, cadavers, CT, radiography), and methodological design.

#### 2.2.6. Statistical Synthesis of Per-Clavicle Prevalence

The overall prevalence of supraclavicular foramen was estimated using a random-effects model with restricted maximum likelihood (REML). Because SCF is a rare anatomical variant, prevalence proportions were variance-stabilized using the Freeman–Tukey double-arcsine transformation prior to pooling. Results were then back-transformed to the original proportion scale for interpretation. Between-study heterogeneity was quantified using Cochran’s Q and the I^2^ statistic, interpreted as the proportion of total variability attributable to true differences rather than sampling error [20]. Subgroup analyses were conducted according to study material (dry clavicles, cadaveric dissections, CT imaging, and radiography).

#### 2.2.7. Statistical Analysis of Conventional Marginal Laterality

For studies reporting separate right and left clavicular counts, conventional laterality was examined using odds ratios (ORs) comparing left versus right clavicles. This analysis treats clavicles as independent units and does not account for within-individual pairing. Pooled ORs were calculated using a random-effects model.

#### 2.2.8. Feasibility-Based Paired Laterality

Several studies reported right- and left-sided counts but did not specify the number of bilateral cases. Because left and right clavicles belong to the same individual, marginal side counts do not uniquely determine the true paired distribution (left-only, right-only, bilateral, neither). To preserve the observed side-specific frequencies while reconstructing the unknown joint distribution, we applied a feasibility-based model parameterized by λ (0≤λ≤1):

$$p_{11}(\lambda )=(1-\lambda )p_{L}p_{R}+\lambda min(p_{L},p_{R})$$

where $p_{L}$ is the prevalence in left clavicles, $p_{R}$ is the prevalence in right clavicles, and $p_{11}$ is bilateral prevalence. Here, λ=0 denotes independence of sides, λ=1 maximal feasible concordance, and λ=0.5 the midpoint assumption. From this reconstructed joint distribution, we derived:

$$p_{10}=p_{L}-p_{11}$$

$$p_{01}=p_{R}-p_{11}$$

where $p_{10}$ is left-only prevalence and $p_{01}$ is right-only prevalence.

The paired odds ratio for unilateral laterality was then defined as:

$$\mathrm{Paired}\;\mathrm{OR}=\frac{p_{10}}{p_{01}}.$$

This quantity compares the probability of left-only versus right-only SCF within individuals. When bilateral counts were explicitly reported, those observed values were used directly and took precedence over reconstruction. When the true body-level denominator was known, it was entered directly; otherwise, the effective paired denominator was conservatively set to the smaller of the right- and left-side sample sizes [21].

Papadatos’ study was excluded from paired laterality because laterality and bilateralism were not reported, despite the paired nature of the cadaveric material [6]. Santos’ study was retained for paired laterality based on side-specific clavicle counts, but its reported bilateral skeletons were not incorporated into the main paired model because they refer only to the identified 80-skeleton subset rather than to the full 450-clavicle mixed sample [17].

Although Parsons (1916) reported two bilateral cases, that study was not eligible for the main bilateral-prevalence model because bilateral occurrence was mentioned within a 286-clavicle series without a directly reported body-level denominator [22].

#### 2.2.9. Main Observed Bilateral Prevalence Model

Observed bilateral prevalence was analyzed separately from the paired laterality reconstruction. Only studies that explicitly reported both the number of examined bodies and the number of bilateral cases were included in the main bilateral-prevalence model. Accordingly, the primary body-level bilateral analysis included four studies [1,23,24,25].

#### 2.2.10. Sensitivity Analyses

Sensitivity analyses were performed at two levels. First, the pooled paired odds ratio was recalculated across the full admissible range of λ (0 to 1) to assess the robustness of the laterality conclusion under different plausible within-individual dependence structures. Second, a separate bilateral-prevalence sensitivity analysis was performed by adding Santos’ study as a distinct data point based on the identified 80-skeleton subset only (3 bilateral cases among 80 individuals) [17]. This sensitivity analysis was kept separate from the main bilateral-prevalence model because the bilateral information from Santos does not correspond to the full 450-clavicle sample used for prevalence and marginal laterality analyses.

#### 2.2.11. Assessment of Small-Study Effects and Reporting Bias

Small-study effects and reporting bias were assessed primarily using the Doi plot and the corresponding Luis Furuya-Kanamori (LFK) index, interpreted according to the standard framework in which transformed study effect sizes are plotted on the x-axis against software-derived absolute ∣Z∣ values reflecting study precision on the y-axis [26,27]. Values of ∣LFK∣ < 1 were interpreted as indicating no asymmetry, 1 ≤ ∣LFK∣ ≤ 2 as minor asymmetry, and ∣LFK∣ > 2 as major asymmetry. Because conventional significance-based tests may be underpowered in meta-analyses with a limited number of studies, Egger’s and Begg’s tests were treated as supplementary rather than primary asymmetry diagnostics. Galbraith and trim-and-fill analyses were additionally performed as secondary sensitivity checks.

The pooled maximum reporting bias, attributable to the prevalence meta-analysis of rare anatomic variants and potentially affected by unregistered or small-sample studies, was quantified using the sample-size adjustment proposed elsewhere [28]. The maximum reporting bias b was calculated as:

$$b=1-\frac{1}{1+y^{y}/y!}$$

where y denotes the number of observed supraclavicular foramina in each study. The standard error of bias (bSE) was calculated as:

$$bSE=\frac{1}{\sqrt{4n+2}}$$

where n denotes study sample size. This expression corresponds to the variance-stabilized approximation commonly used for arcsine-based transformations of binomial proportions and was therefore also applied to Freeman–Tukey-transformed prevalence estimates.

**Table 1:** Characteristics of included studies.

Study	Origin	Type	Explicit Joint Left–Right Information	Total CL	Right CL	Left CL	Total CL with Canal	Right CL with Canal	Left CL with Canal	Total Bodies	Bilateral Canal
Parsons (1916) [22] ¶¶	UK	Dry CL	No	286	NR	NR	7	NR	NR	NR	2
Pühringer (1920) [29]	Austria	Dry CL	No	300	NR	NR	13	NR	NR	NR	NR
Santos (1927) [17]	Portugal	Dry CL	Partially ¶	450	215	235	30	7	23	80 §	3 §
Ahlborg (1931) [30]	Sweden	Dry CL	No	200	NR	NR	6	NR	NR	NR	NR
Skarby (1936) [31]	Germany	Ro imaging	Yes	2000	1000	1000	15	5	10	1000	NR
Olivier (1951) [32]	France	Dry CL	No	170	NR	NR	4	NR	NR	NR	NR
Papadatos (1980) [6]	France	Cadavers	No	508	254	254	10 ⁂	NR	NR	254	NR
Sommerlad (1981) [23]	UK	Cadavers	Yes	188	94	94	1	NR	NR	94	0
Chung (1995) [24]	Korea	Ro imaging	Yes	600	300	300	5	3	2	300	1
Chung (1995) [24]	Korea	Dry CL	No	347 ⁑	166	189	5	3	2	NR	NR
Jelev (2007) [1]	Bulgaria	Dry CL	No	185	94	91	7 ‡	2	5	NR	NR
Jelev (2007) [1]	Bulgaria	Cadavers	Yes	112	56	56	5	2	3	56	1
Nathe (2011) [3] #	USA	Cadavers	No	37	NR	NR	0	0	0	NR	NR
Chanda (2014) [33]	India	Dry CL	No	54	25	29	1	1	0	NR	NR
Natsis (2016) [25]	Greece	Cadavers	Yes	154	77	77	2	0	2	77	0
Natsis (2016) [25]	Greece	Dry CL	No	228	NR	NR	5	NR	NR	NR	NR
Khadanovich (2025) [34]	Czech	Dry CL	No	524	265	259	22 †	9	13	NR	NR
Khadanovich (2025) [34]	Czech	Cadavers	Yes	10	5	5	1	1	0	5	NR
Khadanovich (2025) [34]	Czech	CT imaging	Yes	200	100	100	8	3	5	100	NR
Present study	Greece	Dry CL	No	115	60	55	3	1	2	NR	NR

CL, clavicles; Ro, radiographic; CT, computed tomography; NR, not reported. † A double canal was observed in two cases (right). ‡ A double canal was observed in one case (right). § Santos (1927) [17] body-level information refers only to the identified 80-skeleton subset; the 3 bilateral skeletons were used only in sensitivity analyses and were not assigned to the full 450-clavicle mixed sample. ¶ “Partially” indicates that side-specific clavicle counts were extractable and a paired skeleton subset existed, but bilateral information did not apply to the full mixed sample. ⁂ Papadatos (1980) [6]: the primary paper reported 10 positive individuals among 254 cadavers, but laterality and bilateralism were not reported; accordingly, person-level positivity was not treated as side-specific clavicle-level information. ⁑ Chung (1995) [24] dry-bone series: 355 clavicles were available, but 347 were evaluable for this specific feature in the primary report. # Nathe (2011) [3]: Thirty-seven shoulders, representing 37 clavicles, were dissected, and no transclavicular canals were identified. The study therefore contributed 0/37 to the per-clavicle prevalence analysis. Because donor-level pairing, the number of examined individuals, and explicit bilateral counts were not reported for this endpoint, the study was not included in the paired-laterality or observed bilateral-prevalence analyses. ¶¶ Parsons (1916) [22]: seven perforated clavicles were reported among 286 examined bones, and two cases were described as bilateral; however, the number of examined individuals was not reported, and the author explicitly noted that many observations were based on single recovered clavicles. Accordingly, Parsons was not included in the body-level bilateral-prevalence model.

#### 2.2.12. Tools and Reproducibility

Different estimands required different analytical tools. Rare-variant prevalence was pooled using single-arm proportion methods; conventional left-versus-right marginal laterality used standard comparative meta-analysis of side-specific clavicle counts; paired laterality required custom feasibility-based reconstruction of the unobserved joint left-right distribution in a dedicated R workflow using random-effects models; and observed bilateral prevalence was analyzed separately at the body level using studies with directly extractable bilateral counts. This multi-tool strategy was chosen to match each model to its inferential scale rather than force all outcomes into a single generic framework. The custom paired-analysis code, study-level extraction logic, and sensitivity analyses are available from the corresponding author upon reasonable request. Figures were generated using Stata/BE version 19.0 (StataCorp LLC, College Station, TX, USA), Review Manager (RevMan) version 5.3 (The Nordic Cochrane Centre, The Cochrane Collaboration, Copenhagen, Denmark), or Python version 3.13.5 (Python Software Foundation) with Matplotlib version 3.10.8.

No external financial support was received. The review was performed in accordance with a prespecified protocol. Registration in PROSPERO was not feasible because prevalence meta-analyses are currently not eligible for registration.

## 3. Results

### 3.1. Prevalence, Size, and Location of Supraclavicular Foramina in the Present Observational Study

**Figure 2:**
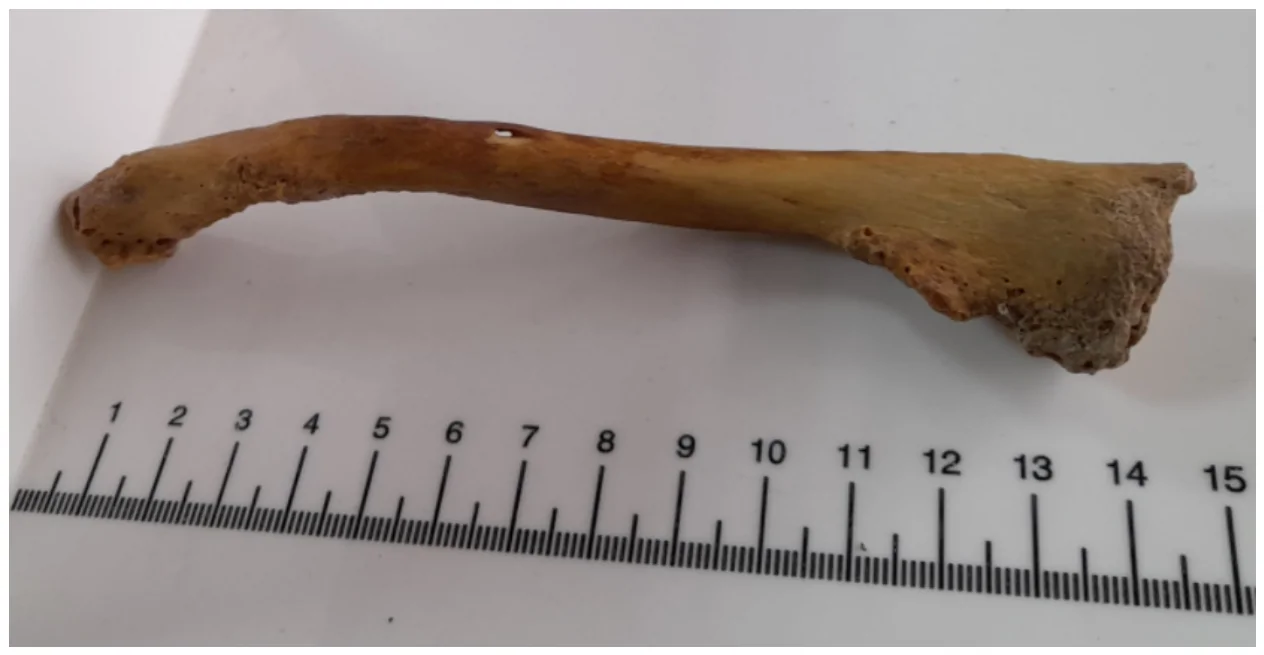
Supraclavicular foramen.

A total of 115 dry clavicles (60 right and 55 left) were examined. Complete transclavicular canals, each identified by two external openings connected by a complete transosseous passage, were present in 3 of 115 clavicles (2.6%) (Figure 2). Two affected clavicles were located on the left side and one on the right. The detected canals had a mean aggregate transverse wire span of 1.5 ± 0.8 mm (population SD), a mean lateral-distance/total-length ratio of 0.441 ± 0.012, and corresponding mean absolute distances of 60.0 ± 5.2 mm from the acromial end and 73.4 ± 9.5 mm from the sternal end. The mean inter-opening distance, used as canal length, was 5.9 ± 2.4 mm. Using the projected-obliquity calculation described in the Section 2, the canals showed a mean projected obliquity of 24.3 ± 8.8°, indicating a non-perpendicular course across the bone. Caliper-based measurements further showed that the two external openings were typically oval, indicating that wire-based patency estimates and external-opening dimensions captured complementary aspects of canal morphology.

**Figure 3:**
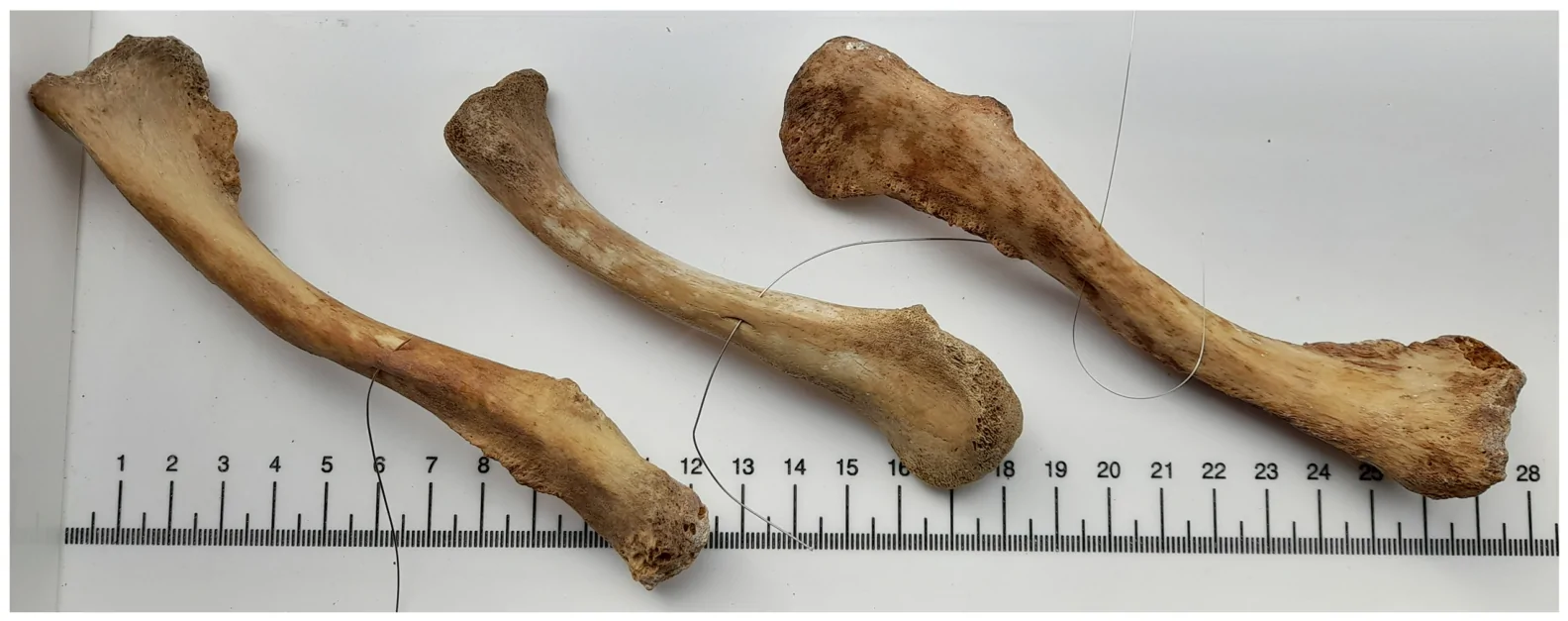
Three clavicles showing complete transclavicular canals identified by two external openings connected by a complete transosseous passage. The right-sided canal was markedly narrower; the largest individual wire that traversed it measured 0.2 mm.

In both left clavicles, a stainless-steel wire of ≥0.4 mm in diameter could be passed easily through the canal, confirming complete transosseous patency. In contrast, in the right clavicle, passage was possible only with a 0.2 mm wire, suggesting a much narrower canal (Figure 3).

Because only three complete canals were identified, formal normality testing and inferential comparisons of morphometric variables were not performed; individual measurements are provided in Supplementary Table S2, and mean ± standard deviation values are presented as descriptive summaries.

Inter-rater reliability was excellent, with intraclass correlation coefficients (ICCs) of 0.999 (95% CI: 0.996–1.000) for external opening measurements, 0.988 (95% CI: 0.957–0.998) for acromial distance, and 0.986 (95% CI: 0.950–0.998) for sternal distance.

### 3.2. Meta-Analysis

#### 3.2.1. Study Selection

**Figure 4:**
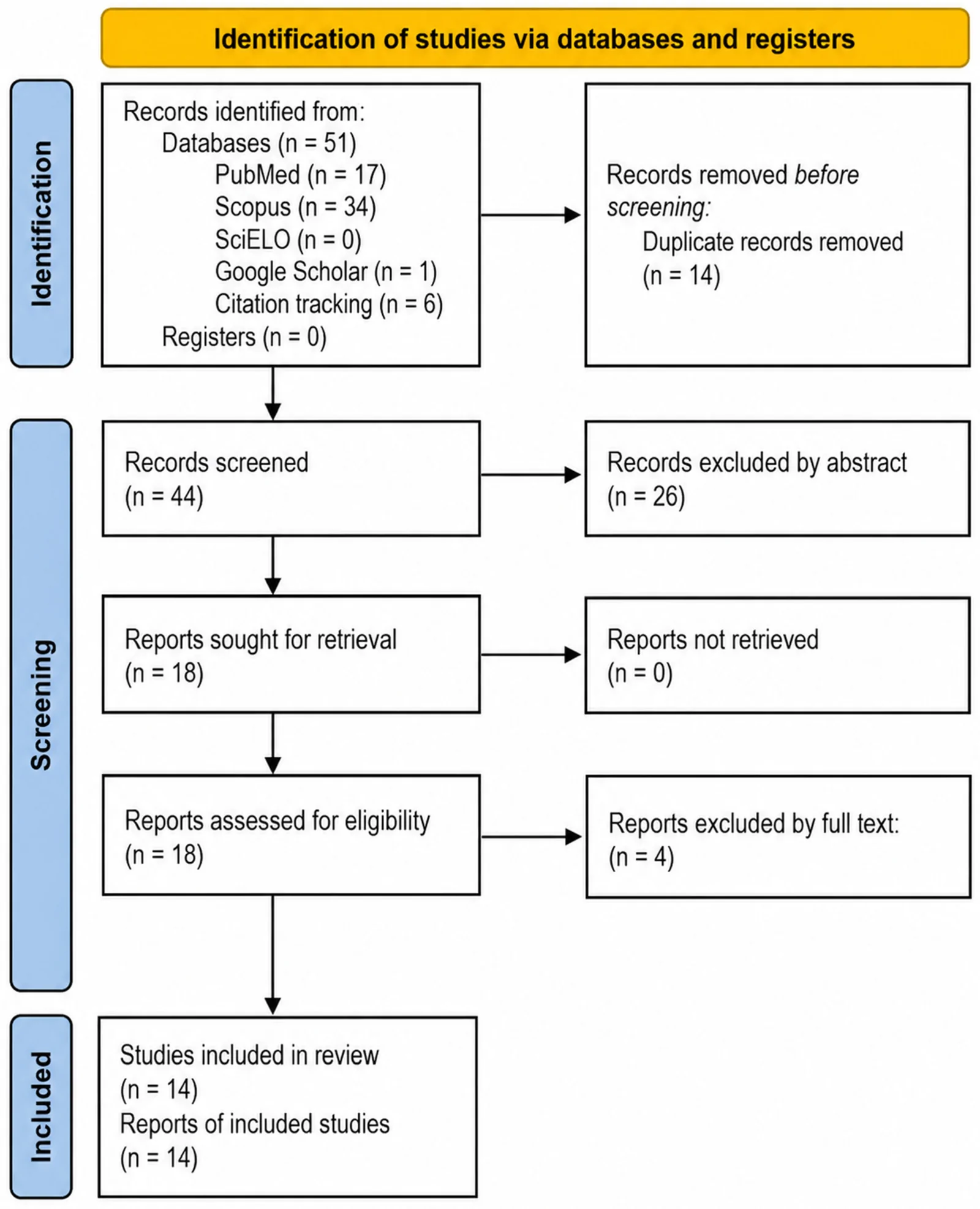
PRISMA flow diagram.

Overall, 58 records were identified: 51 through bibliographic database searches (PubMed, *n* = 17; Scopus, *n* = 34; SciELO, *n* = 0), one through Google Scholar, and six through backward and forward citation tracking. After removal of 14 duplicates, 44 records underwent title and abstract screening, of which 26 were excluded as irrelevant. Eighteen reports were sought and retrieved for full-text assessment; four were excluded for the reasons presented in Supplementary Table S1, leaving 14 studies for qualitative and quantitative synthesis (Figure 4).

The characteristics of the included studies, together with the availability of side-specific and body-level information relevant to each inferential level, are summarized in Table 1.

#### 3.2.2. Quality Assessment

Using the Anatomical Quality Assessment (AQUA) tool, Domain 1 (Objectives and subject characteristics) showed the greatest vulnerability to bias, primarily because many studies lacked information on cadaveric sex, age, and medical background. The remaining domains—Study Design, Methodological Characterization, Descriptive Anatomy, and Reporting of Results—were generally more robust. The study-level AQUA judgments are presented in Table 2.

To distinguish directly observed specimen-level findings from reconstructed individual-level outcomes, the meta-analytic results were organized into four inferential levels: per-clavicle prevalence, conventional marginal laterality, feasibility-based paired laterality, and observed body-level bilateral prevalence. These analyses are complementary rather than interchangeable because they address different anatomical questions and operate on different sampling scales.

#### 3.2.3. Morphometric Characteristics of Supraclavicular Canals

Morphometric data were too sparse and definitionally heterogeneous for formal meta-analysis; Table 3 therefore presents descriptive synthesis only.

Across the available morphometric datasets, the canal-count-weighted descriptive mean medial distance from the sternal end was 69.7 ± 10.6 mm, the mean lateral distance from the acromial end was 60.8 ± 11.5 mm, and the mean acromial ratio was 0.440 ± 0.063. The weighted descriptive mean canal length was 6.4 ± 2.3 mm. For the two modern studies reporting transverse canal-caliber measures, the exploratory canal-count-weighted descriptive mean was 1.94 ± 0.63 mm. Because the measurement protocols differed, this value should be interpreted cautiously. In a sensitivity analysis that downweighted the six bilaterally paired Santos canals using an effective-sample-size correction assuming *r* = 0.75, these descriptive means changed only trivially (lateral distance 60.8 mm, acromial ratio 0.441, canal length 6.3 mm).

#### 3.2.4. Pooled Per-Clavicle Prevalence

**Figure 5:**
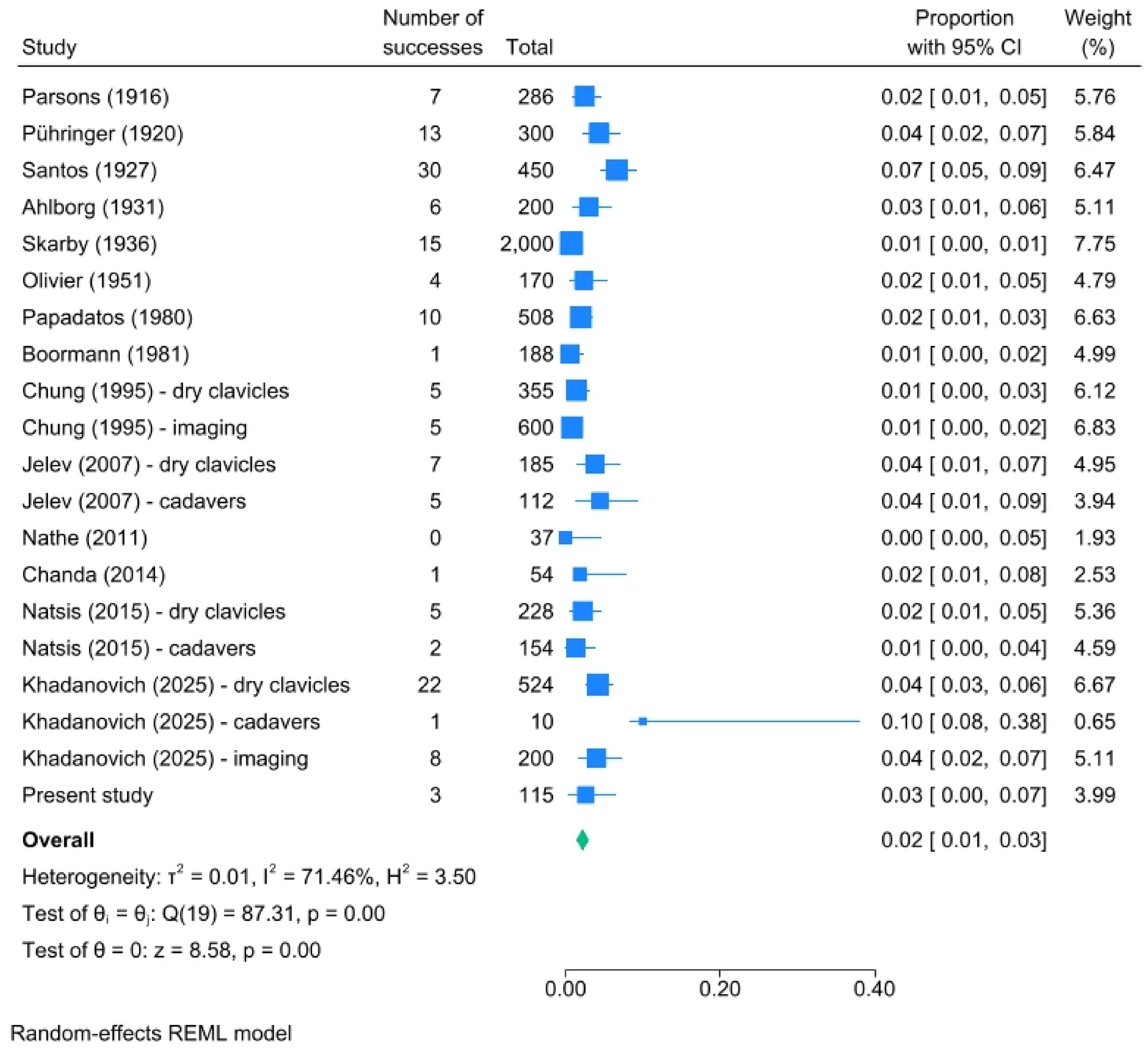
Pooled prevalence of supraclavicular foramen (2.2%; 95% CI: 1.4–3.0%; I^2^ = 71%); study labels correspond to the included reports listed in Table 1 and the reference list. Study-specific data were derived from the included reports [1,3,6,17,22,23,24,25,29,30,31,32,33,34].

The pooled analysis showed that supraclavicular foramen was present in 2.2% of clavicles (95% CI: 1.4–3.0%; I^2^ = 71%) (Figure 5).

**Figure 6:**
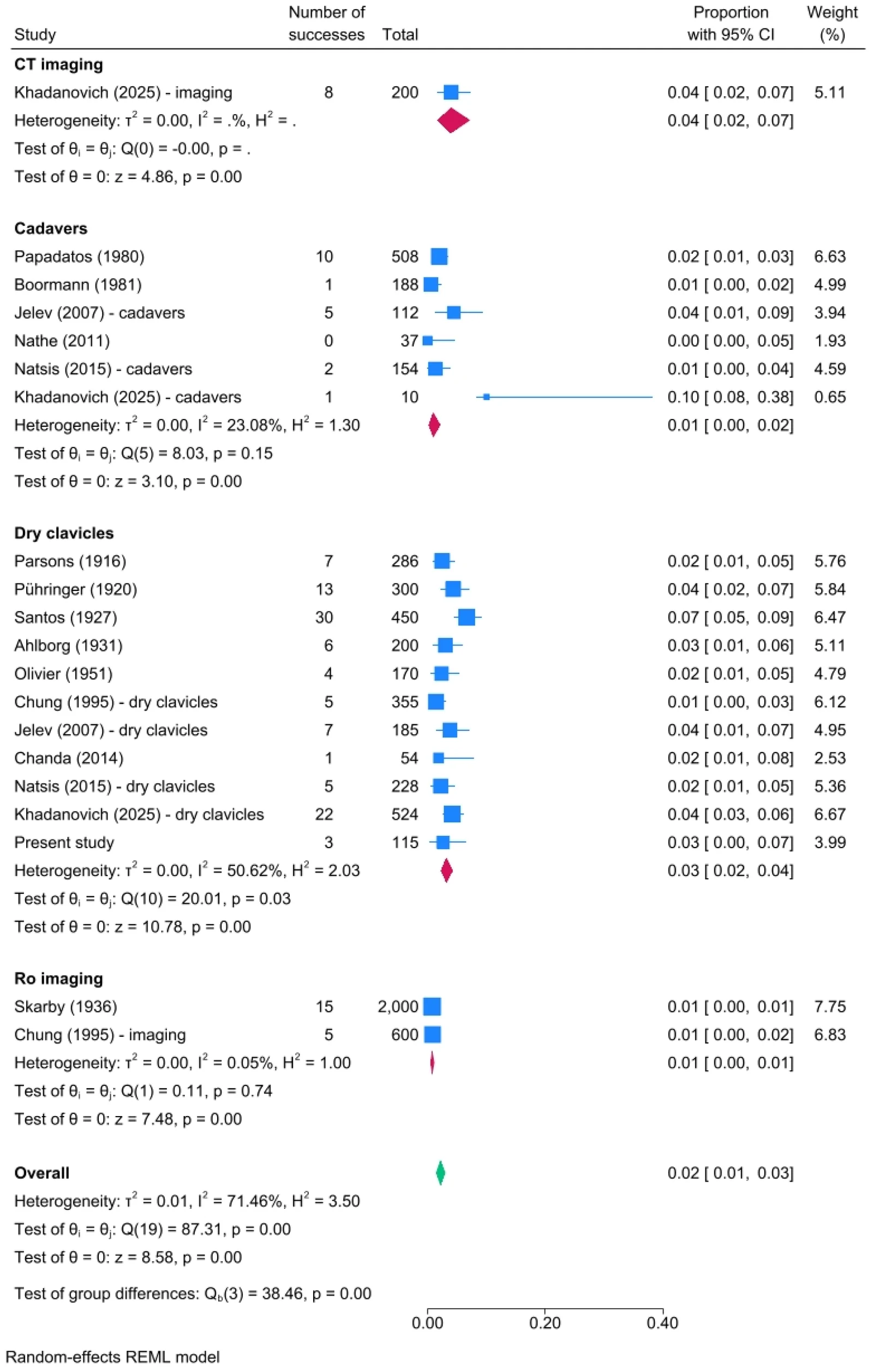
Subgroup analysis of supraclavicular foramen prevalence according to study material. In the CT subgroup, heterogeneity: not estimable (single study). Study-specific data were derived from the included reports [1,3,6,17,22,23,24,25,29,30,31,32,33,34].

Subgroup analysis demonstrated significant differences (*p* < 0.001) in prevalence according to study material, with pooled estimates of 4.0% (95% CI: 1.7–7.2%) for CT studies, 1.0% (95% CI: 0.2–2.1%) for cadaveric studies, 3.2% (95% CI: 2.3–4.3%) for dry-bone studies, and 0.8% (95% CI: 0.4–1.1%) for radiographic studies (Figure 6).

#### 3.2.5. Pooled Conventional Marginal Laterality

**Figure 7:**
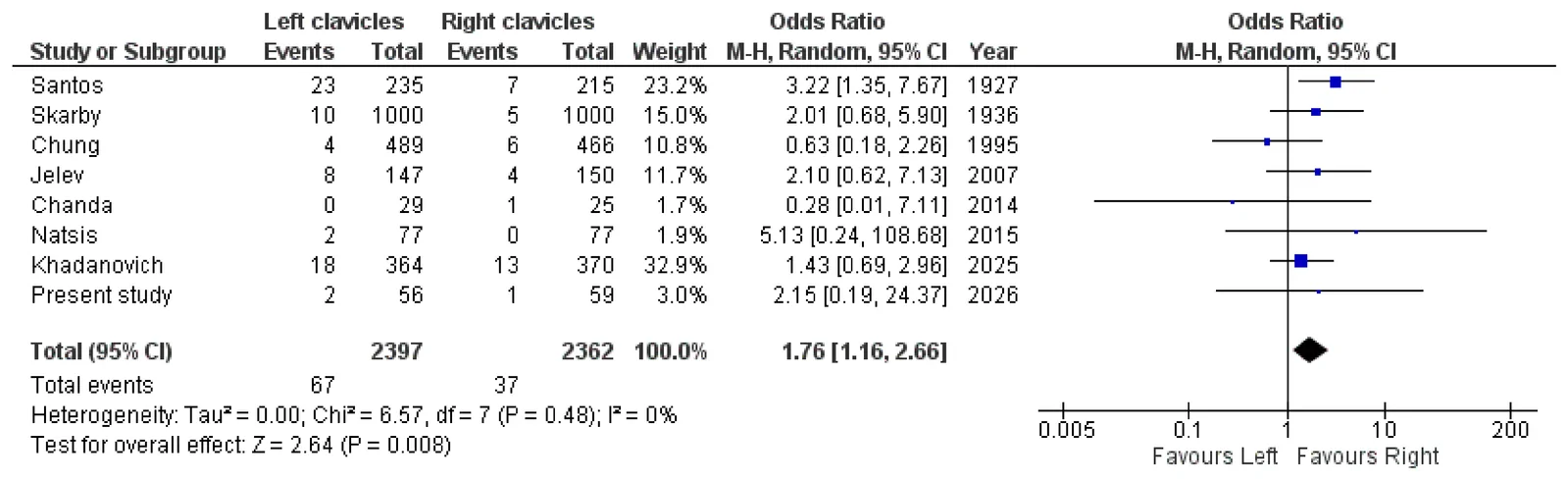
Forest plot of the pooled odds ratio (OR) for the presence of a supraclavicular foramen in the left versus right clavicle. Squares represent study-specific ORs, with their size reflecting study weight; horizontal lines represent 95% confidence intervals. The diamond represents the pooled random-effects estimate (OR = 1.76; 95% CI: 1.16–2.66), and the vertical reference line denotes the null value (OR = 1). Values greater than 1 indicate left-sided predominance. Study-specific data were derived from the included reports [1,17,24,25,31,33,34].

For studies reporting separate right- and left-sided clavicular counts, conventional marginal laterality analysis showed a significant left-sided predominance. The pooled odds ratio for the presence of a supraclavicular foramen in left versus right clavicles was 1.76 (95% CI: 1.16–2.66; I^2^ = 0%) (Figure 7). This analysis reflects the conventional clavicle-level comparison and does not account for within-individual pairing.

#### 3.2.6. Paired Laterality

**Figure 8:**
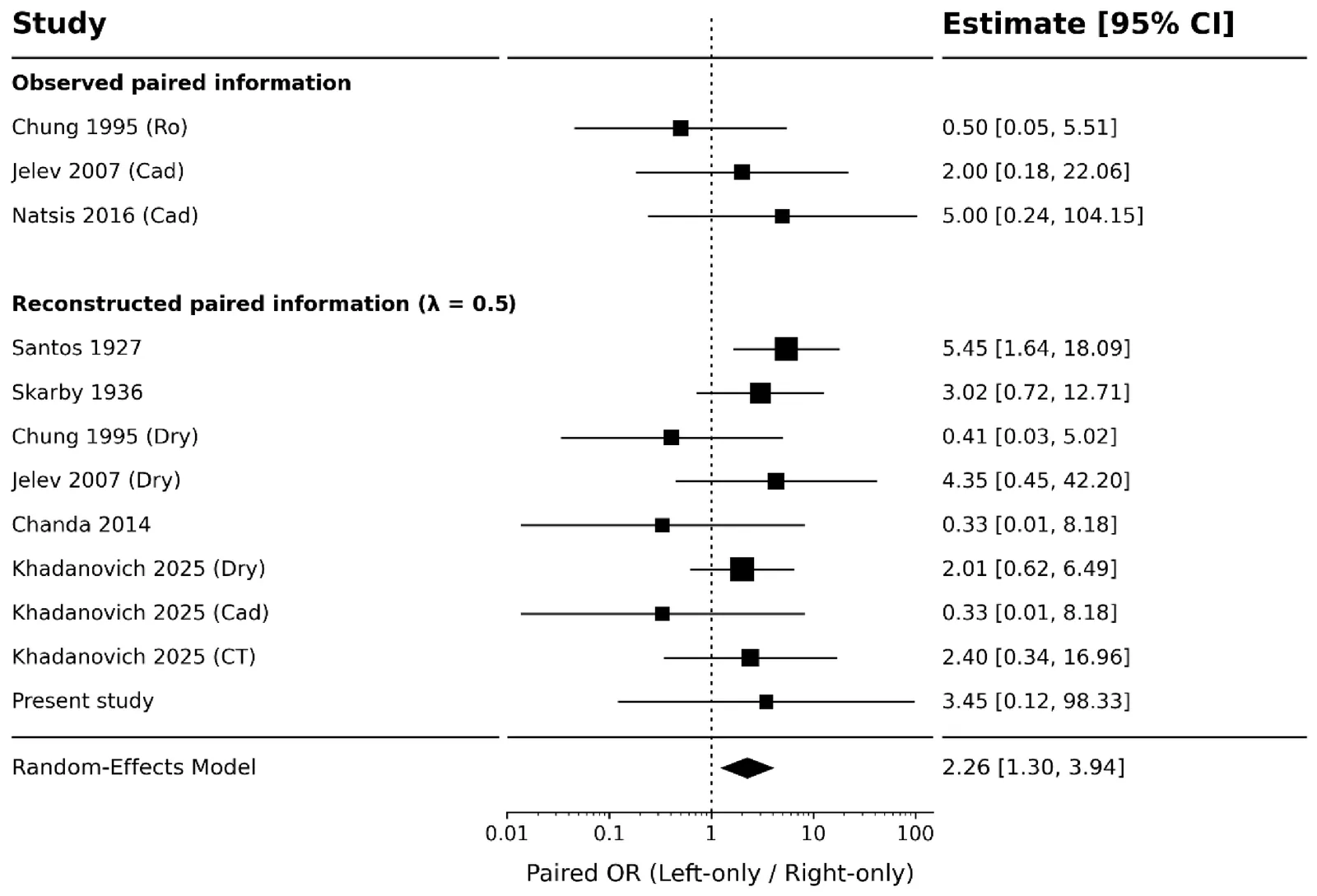
Feasibility-based paired laterality analysis of supraclavicular foramen. Forest plot of the pooled paired odds ratio (OR) for left-only versus right-only occurrence. Squares represent study-specific paired ORs, horizontal lines represent the corresponding 95% confidence intervals, and the diamond represents the pooled random-effects estimate. The vertical dotted line marks the null value (OR = 1). Studies with directly reported bilateral counts are shown separately from studies requiring reconstruction; reconstruction was applied only when bilateral counts were unavailable, using the midpoint feasibility assumption (λ=0.5). Values greater than 1 indicate a greater probability of left-only than right-only SCF. Study-specific data were derived from the included reports [1,17,24,25,31,33,34].

In the feasibility-based paired analysis, the pooled odds ratio for left-only versus right-only occurrence was 2.26 (95% CI: 1.30–3.94), indicating that unilateral SCF was more than twice as likely to occur on the left side as on the right (Figure 8). This estimate incorporated known body-level denominators and observed bilateral counts where available, while reconstructing the bilateral joint distribution only for studies lacking direct bilateral information. Between-study heterogeneity for the paired laterality effect was minimal.

#### 3.2.7. Main Observed Bilateral Prevalence

**Figure 9:**
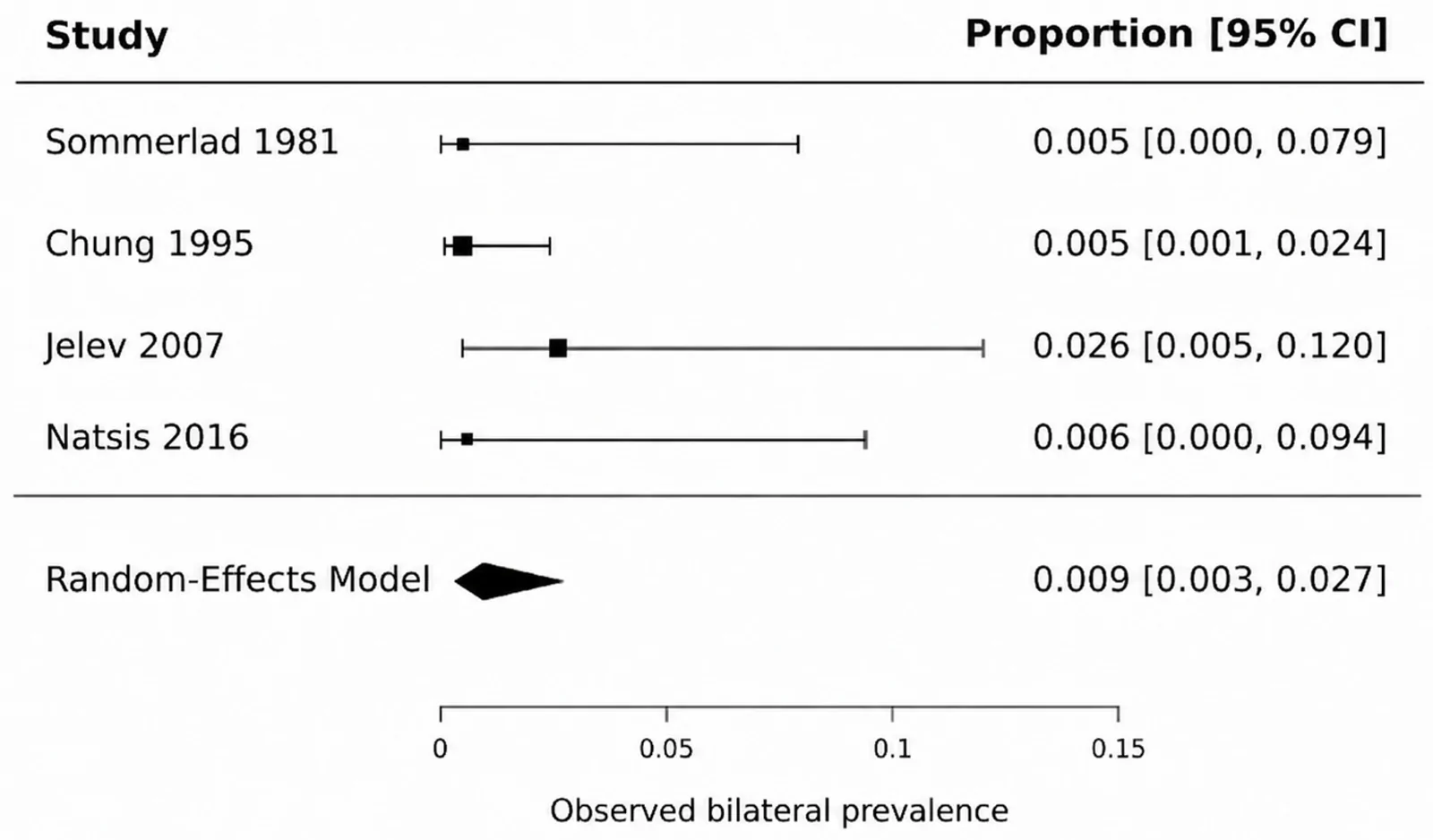
Forest plot of the observed bilateral prevalence of supraclavicular foramen at the individual level. Squares represent study-specific prevalence estimates, horizontal lines indicate the corresponding 95% confidence intervals, and the diamond represents the pooled estimate obtained using the primary logit random-effects model with continuity correction. Study-specific data were derived from the included reports [1,23,24,25].

The main observed bilateral-prevalence model, restricted to studies with directly reported body-level bilateral data, yielded a pooled bilateral prevalence of 0.95% (95% CI: 0.33–2.70%) under the primary logit random-effects model (Figure 9).

A supplementary reanalysis of the same four datasets using the Freeman–Tukey double-arcsine transformation yielded a lower pooled estimate (0.14%, 95% CI: 0.00–0.87%). Because the inverse Freeman–Tukey transformation is sample-size dependent and may be unstable in sparse single-proportion meta-analysis, the logit-based estimate was retained as the primary result.

Parsons (1916) was not entered into this model despite reporting two bilateral cases, because the denominator was reported only as 286 clavicles rather than as a defined number of examined bodies [22]. Nathe (2011) was not entered into the observed bilateral-prevalence model because the study reported 37 examined shoulders but did not provide an identifiable body-level denominator or an explicit bilateral count for the transclavicular-canal endpoint [3].

#### 3.2.8. Bilateral Sensitivity Analysis

Adding the identified 80-skeleton subset from Santos (1927) [17] in a separate sensitivity analysis (3 bilateral cases among 80 individuals) increased the pooled observed bilateral prevalence from 0.95% (95% CI: 0.33–2.70%) to 1.52% (95% CI: 0.53–4.26%), without changing the conclusion that bilateral SCF is uncommon. This analysis was kept separate because the bilateral data from Santos apply only to the identified paired subset, not to the full 450-clavicle mixed sample [17].

#### 3.2.9. Sensitivity Analysis for λ

**Figure 10:**
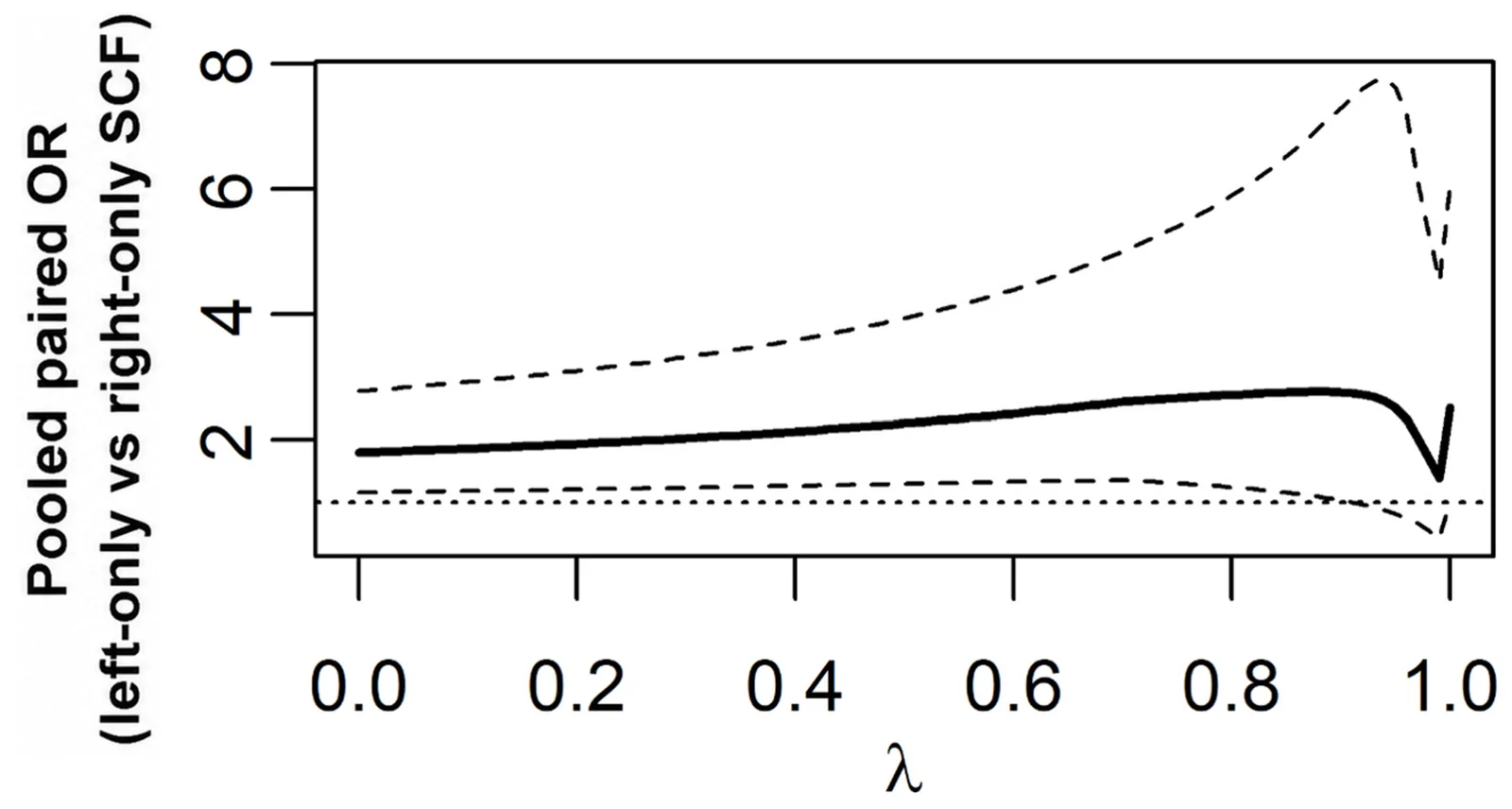
Sensitivity of the pooled paired laterality estimate to the assumed within-individual dependence parameter. The ordinate shows the pooled paired odds ratio (OR) for left-only versus right-only supraclavicular foramen, and the abscissa shows the feasibility parameter λ, ranging from 0 (independence) to 1 (maximal feasible concordance) for studies lacking directly observed bilateral counts. Studies with observed bilateral counts were held fixed. The solid curve represents the pooled OR, the dashed curves represent the 95% confidence interval, and the horizontal dotted line marks the null value (OR = 1). The pooled estimate remained greater than 1 across the admissible range, supporting persistent left-sided predominance, although boundary instability occurred near λ=1 as the reconstructed discordant counts approached zero.

Across the admissible range of λ, the pooled paired odds ratio remained consistently greater than 1, indicating persistent left-sided predominance under all feasible dependence structures for studies lacking explicit bilateral counts (Figure 10). Near λ = 1, the curve showed the expected boundary instability associated with vanishing discordant counts under strong concordance, but this did not reverse the direction of the effect. Thus, the qualitative conclusion of left-sided unilateral predominance was robust to the dependence assumption.

#### 3.2.10. Results of the Small-Study-Effect and Reporting-Bias Assessment

**Figure 11:**
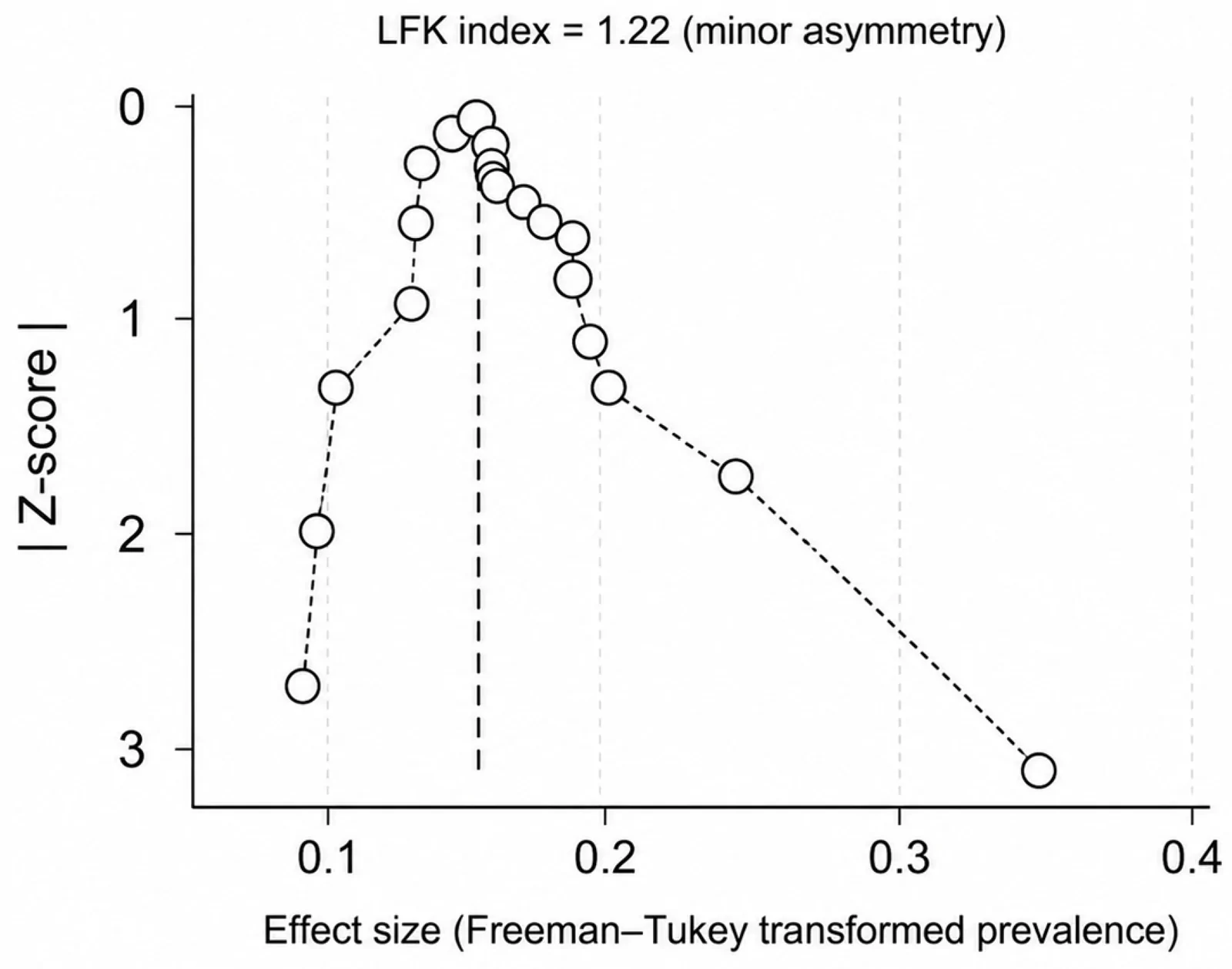
Doi plot and Luis Furuya-Kanamori index for the per-clavicle prevalence meta-analysis. Each circle represents one study. The abscissa shows the study-specific effect size expressed as Freeman–Tukey transformed prevalence, whereas the ordinate shows the software-derived absolute ∣Z∣ value used as the folded representation of study precision. The vertical dashed line marks the effect-size reference around which symmetry is assessed. The Luis Furuya-Kanamori index was 1.22, which falls within the predefined range for minor asymmetry. The unequal distribution of studies around the reference line therefore suggests possible small-study effects but should not be interpreted as definitive evidence of publication bias, particularly given the limited number of studies and substantial between-study heterogeneity.

Evidence of small-study effects was suggested by the Doi plot, which showed minor asymmetry with an LFK index of 1.22 (Figure 11). In the present prevalence meta-analysis, the Doi plot/LFK framework was considered the primary asymmetry assessment, whereas Egger’s and Begg’s tests were interpreted as supplementary because of their limited power in meta-analyses with relatively few studies.

Supplementary visual diagnostics provided mixed evidence. The Galbraith plot showed some study-level deviations from the fitted relationship (Supplementary Figure S2), and the funnel plot displayed visible asymmetry; however, the trim-and-fill procedure did not impute any additional studies and therefore did not alter the pooled estimate (Supplementary Figure S3). Egger’s and Begg’s tests were also non-significant (*p* = 0.281 and *p* = 0.846, respectively). Accordingly, the overall pattern was interpreted as suggestive, but not definitive, evidence of small-study effects.

The maximum reporting bias was estimated at 6% (95% CI: 2–15%; I^2^ = 100%) (Supplementary Figure S4).

#### 3.2.11. Critical Appraisal of the Present Meta-Analysis

Application of the Critical Appraisal Tool for Anatomical Meta-Analyses (CATAM) was performed independently by four reviewers (V.P., V.T., A.N., and A.M.). All four raters assigned identical CATAM scores across all domains, indicating complete exact agreement; therefore, no adjudication or consensus rescoring was required. The final CATAM scores for the present study were as follows: Title, 2 points; Abstract, 4 points; Introduction, 6 points; Methods, 14 points (Search strategy = 4; Selection criteria = 2; Data extraction = 2; Quality assessment = 4; Statistical analysis = 2); Results, 12 points (Search results = 2; Characteristics of included studies = 2; Outcomes = 8); Discussion, 6 points; Conclusion, 4 points; References, 2 points.

## 4. Discussion

### 4.1. Principal Findings and Comparison with Previous Literature

The present study contributes at two complementary levels. In the observational component, complete transclavicular canals were uncommon and occupied a reproducible mid-to-lateral region of the clavicle, consistent with the general pattern described in previous osteological, cadaveric, and imaging studies. At the meta-analytic level, the pooled evidence confirmed a low per-clavicle prevalence, supported a predominance on the left side, and indicated variation according to study material. Detection was higher in dry-bone and computed-tomography studies than in cadaveric and plain-radiographic series. These differences should not necessarily be interpreted as biological variation alone, because ascertainment is strongly influenced by the material and technique used. Complete osseous canals are readily recognizable in dry bone and CT, whereas shallow grooves or incompletely ossified fibro-osseous passages may be overlooked, particularly on plain radiographs or during dissections not specifically directed toward this variant [34].

The findings also demonstrate that apparently similar anatomical counts may represent different inferential quantities. Per-clavicle prevalence estimates the frequency of the variant in individual bones; marginal laterality compares observed left- and right-sided frequencies without accounting for donor-level pairing; paired laterality compares left-only and right-only occurrence within individuals; and bilateral prevalence measures the proportion of individuals affected on both sides. These estimands are complementary rather than interchangeable, and their separation is particularly important in historical and osteological datasets in which donor-level provenance is incomplete.

### 4.2. Morphological and Developmental Interpretation

The structure considered here is not currently included in the Terminologia Anatomica, and the literature has used several overlapping expressions, including “foramen for the supraclavicular nerve” [31]. In the present study, transclavicular canal denotes a complete osseous passage, whereas supraclavicular foramen denotes either external opening and is also used as the preferred general term for the variant. A groove is considered an incomplete canal without a fully ossified roof. This distinction is useful but not absolute, because an osseous groove may be converted functionally into a fibro-osseous tunnel by a bridging fibrous band. Grooves, partial canals, and complete canals may therefore represent related configurations along the same anatomical spectrum [1,29,34,35].

Distinguishing a transclavicular canal from the ordinary clavicular nutrient foramen is clinically and radiologically important. Olivier described a true traversing passage extending from the posterior border toward the anterior surface near the junction of the middle and lateral thirds and noted that such canals could be overlooked or confused with nutrient foramina [32]. Parsons similarly observed that the supraclavicular perforation could share a posterior opening with the nutrient foramen while continuing as a distinct transosseous passage [22]. A specimen from the present collection illustrates this distinction by showing a complete supraclavicular canal together with separate blind-ending nutrient foramina (Supplementary Figure S5). The broader term “neurovascular foramina” should therefore be used cautiously, because it may conflate the rare transclavicular canal with the much more frequent vascular nutrient canal [36].

Canal direction may provide a practical discriminator. Unlike a blind-ending nutrient canal, a complete transclavicular canal has two communicating external openings and generally follows an oblique posterior-to-superior or posterior-to-anterior course [1,31]. The projected orientation recorded in the present specimens was consistent with this directional pattern. A further differential diagnosis is the congenital variant reported by Viciano et al., in which much larger medial diaphyseal foramina and an associated canal were attributed to vascular inclusion during ossification. That anomaly differs in size, location, and presumed developmental origin from the smaller and more lateral variant considered here [37].

The most widely accepted developmental explanation is that a normally superficial supraclavicular nerve branch becomes incorporated into the mesenchymal condensation of the developing clavicle and is subsequently surrounded by bone. This mechanism is anatomically plausible because the clavicle ossifies early and combines intramembranous and endochondral processes. Nevertheless, it remains an inferred mechanism rather than one demonstrated in the present dry-bone specimens. The current observations establish a complete osseous passage but cannot identify its original contents. The canal should therefore be described as presumably or potentially transmitting a supraclavicular branch rather than as directly demonstrating an intraosseous nerve [6,25].

The available morphometric evidence supports—but does not prove—an association with the intermediate supraclavicular branch. Khadanovich et al. reported a mean canal width of 2.0 ± 0.6 mm, measured digitally on calibrated photographs of dry clavicles [34]. In the present study, the mean aggregate transverse wire span was 1.5 ± 0.8 mm and represented an operational lower-bound estimate of the wider internal lumen dimension. Because these methods assess related but non-equivalent aspects of canal caliber, the values were not regarded as strictly interchangeable. Their canal-count-weighted combination in Table 3 is therefore presented only as an exploratory descriptive summary rather than as a formal pooled estimate. Nevertheless, their broadly similar millimetric scale is anatomically consistent with reported diameters of major supraclavicular nerve branches, including approximately 1.1 mm for the medial branch, 1.2 mm for the intermediate branch, and 1.6 mm for the lateral branch [38], as well as the previously reported range of 1–2 mm [23]. This dimensional concordance supports the plausibility of transmission of a supraclavicular branch, particularly the intermediate branch, but does not establish the contents of canals observed in dry bone.

Taken together, the convergence of canal position, orientation, and dimensions across independent historical and modern series indicates that the supraclavicular foramen is not a random osseous perforation. Rather, it appears to represent a recurrent developmental configuration of the mid-to-lateral clavicle, most plausibly related to the course of the intermediate supraclavicular branch. Santos described a generally posterior-to-anterior and medial-to-lateral canal orientation, with the canal/orifice complex situated at a reproducible distance from the acromial end [17]. Olivier similarly placed the perforation near the junction of the middle and lateral thirds [32], whereas Papadatos described true intraosseous canals predominantly in the central clavicle or near the transition between its middle and outer thirds [6]. Modern observations from Chanda, Khadanovich et al., and the present study occupy a comparable topographic corridor [33,34], which also corresponds broadly to the reported crossing region of the intermediate supraclavicular branch [38]. This cross-study concordance supports a structured developmental association rather than an incidental aperture, although the original canal contents cannot be established from dry-bone morphology alone.

The right-sided canal in the present sample represents an important qualification. Its maximum wire-defined patency was only 0.2 mm, whereas reported supraclavicular nerve branches generally measure at least approximately 0.5 mm [39]. Transmission of a typical branch through this particularly narrow passage therefore appears less likely. However, wire-defined internal patency is not equivalent to external foraminal width, and postmortem or developmental narrowing cannot be excluded. Because no soft tissues were available, the passage may represent an unusually narrow supraclavicular canal or another complete transosseous variant.

Historical and modern morphometric data also differ in definitions, measurement landmarks, and reporting completeness. Some studies measured the nearest orifice, others the canal center, and others the distance to a canal margin. Historical summary statistics required reconstruction, and within-individual pairing could not always be identified. The apparent consistency of the mid-to-lateral corridor should consequently be interpreted as a descriptive anatomical pattern rather than as a formally pooled morphometric estimate.

### 4.3. Clinical and Radiological Implications

When a supraclavicular branch passes through a rigid osseous canal, it loses the mobility normally provided by superficial subcutaneous tissue and may become vulnerable to compression, traction, or irritation. Direct anatomical support comes from cases in which the intermediate branch was demonstrated within a clavicular canal [35]. Clinical consequences remain uncommon, however, and the presence of a canal should not itself be regarded as proof of neuropathy. Symptoms are more likely to arise when the canal is affected by trauma, fracture displacement, callus formation, periosteal thickening, repetitive mechanical stress, or other changes in clavicular contour [1,10,25].

Potential manifestations include localized clavicular or anterior shoulder pain, paresthesia, and numbness over the deltoid and upper thoracic regions. Sensory overlap among supraclavicular branches may partially mask injury, so the absence of a sharply demarcated sensory deficit does not exclude clinically relevant irritation [3]. Other anatomical structures can produce similar symptoms. A supraclavicularis proprius muscle has been reported to form a tunnel around the nerve [40], and post-fracture callus may entrap an otherwise normally positioned supraclavicular branch; surgical neurolysis relieved symptoms in the two cases reported by Jupiter and Leibman [41].

The practical relevance of the variant concerns recognition and risk avoidance. On radiographs or CT, a complete transclavicular canal should be distinguished from a nutrient foramen, vascular anomaly, lytic lesion, or post-traumatic defect. During clavicular exposure, plating, and fracture fixation, an atypical transosseous nerve course may place a branch directly within the operative field. Giddie et al. described such a branch during clavicular fixation; division was required for plate placement and was followed by postoperative numbness [7]. Preoperative identification may therefore assist surgical planning and patient counseling.

Knowledge of the variant may also be relevant to regional anesthesia and to the assessment of otherwise unexplained supraclavicular neuralgia. Nathe et al. demonstrated considerable variability in the course of the supraclavicular branches between the medial and lateral clavicular safe zones [3]. Although those safe zones do not specifically predict a transosseous course, awareness of the possibility may help explain incomplete blockade, unexpected sensory symptoms, or nerve injury during procedures near the clavicle. When a symptomatic osseous tunnel is demonstrated and conservative treatment fails, surgical decompression may be beneficial, as reported by Omokawa et al. [10]. These applications concern recognition of a potential risk substrate rather than evidence that every canal is symptomatic.

### 4.4. Laterality and Methodological Implications

The predominance of supraclavicular foramina on the left side is supported by both the conventional marginal comparison and the feasibility-based paired analysis. Its biological explanation remains uncertain. One possibility is asymmetric postnatal remodeling: greater habitual loading of the dominant right upper limb could promote cortical remodeling and closure of small developmental passages, whereas reduced loading on the left may favor their persistence [42]. This interpretation remains speculative because hand dominance and occupational loading were not reported in most source studies.

A developmental or genetic contribution is also plausible. Voisin noted the occurrence of the foramen in fetal and young skeletal material and highlighted its unusually high frequency in Lapita-period Oceanian populations, where founder effects or population isolation may have increased the expression of a genetically influenced trait [43]. Genetic predisposition and biomechanical remodeling need not be mutually exclusive. A developmentally determined canal may persist, narrow, or disappear according to subsequent growth and side-specific mechanical loading.

The analytical findings have implications beyond SCF. Marginal left-versus-right comparisons treat bones as independent and answer whether the variant is more frequently observed on one side at the clavicle level. Paired analysis instead compares discordant individuals—left-only versus right-only cases—and requires knowledge of the joint left–right distribution. Because many studies reported only marginal side counts, part of the paired analysis depended on explicit reconstruction of missing bilateral information. The midpoint estimate should therefore be interpreted as feasibility-based rather than directly observed. Nevertheless, the direction of the laterality effect remained leftward across the admissible dependence range, suggesting that the qualitative finding was not driven solely by the selected midpoint assumption.

Directly observed bilateral prevalence was analyzed separately because it requires both an individual-level denominator and an explicit bilateral count. This distinction prevented reports with clavicle-level denominators or incompletely linked skeletal material from being treated as though they supplied fully paired observations. The resulting framework illustrates why the statistical model must be matched to the anatomical unit of analysis rather than selected solely according to the numerical format of the reported data.

### 4.5. Strengths and Limitations

A principal strength of the study is the integration of direct osteological observation with a systematic synthesis of historical, cadaveric, radiographic, and CT evidence. The analysis explicitly separated four inferential levels, distinguished observed from reconstructed bilateral information, and evaluated the sensitivity of paired estimates to alternative dependence assumptions. The morphometric synthesis also preserved historical evidence while clearly identifying reconstructed and non-comparable measurements.

Several limitations should nevertheless be acknowledged. The observational sample was derived from a single institutional osteological collection, and information on donor age, sex, occupation, hand dominance, and individual left–right pairing was unavailable. Only three complete canals were identified, precluding meaningful assessment of distributional form and limiting the stability and generalizability of the morphometric and inter-rater reliability estimates. Means and standard deviations should therefore be interpreted as descriptive summaries rather than as evidence of normally distributed measurements. Actual canal contents could not be confirmed in dry bone. In addition, discrete wire gauges provided an interval-based estimate of internal patency, whereas caliper measurements characterized external opening dimensions; these measurements describe different aspects of canal morphology and should not be interpreted interchangeably.

At the review level, the included studies were heterogeneous in population, material, detection method, anatomical definition, and reporting quality. Historical reports frequently lacked complete side-specific denominators, donor-level linkage, or standardized morphometric landmarks. Some morphometric values required reconstruction from ranges, and the paired laterality analysis required explicit assumptions when bilateral counts were unavailable. Although sensitivity analyses assessed the influence of these assumptions, they cannot replace directly reported individual-level data.

Transformation choice was consequential in the body-level bilateral analysis because only four datasets were available and several contained zero events. The Freeman–Tukey double-arcsine sensitivity analysis produced a lower estimate than the primary logit model. The logit estimate was retained as primary because it avoids the sample-size-dependent inverse transformation and is more directly interpretable in this sparse-data setting. The difference between models should nevertheless be considered when interpreting the precision of the bilateral-prevalence estimate.

Assessment of small-study effects was constrained by the limited and heterogeneous evidence base. Although the Doi plot/LFK index indicated minor asymmetry and the funnel plot was visually asymmetric, Egger’s and Begg’s tests were non-significant, and the trim-and-fill procedure did not impute any additional studies or modify the pooled estimate. Given the limited number of studies and substantial heterogeneity, these diagnostics were interpreted cautiously, and the overall evidence was considered suggestive rather than definitive for small-study effects or publication bias [27]. Collectively, these limitations reinforce the need for future studies to report standardized canal definitions, raw morphometric measurements, complete side-specific denominators, donor-level pairing, and explicit bilateral occurrence.

## 5. Conclusions

Complete transclavicular canals were uncommon in the present osteological sample and occupied a reproducible mid-to-lateral clavicular position. The meta-analysis estimated a low overall per-clavicle prevalence and supported left-sided predominance in both marginal and feasibility-based paired analyses, although incomplete donor-level pairing and reconstructed bilateral information require cautious interpretation. Directly observed bilateral occurrence was rare. Recognition of this variant may assist surgical planning and radiological interpretation, while the analytical framework illustrates the importance of matching bilateral anatomical data to the appropriate unit of analysis.

## Figures and Tables

**Table 2:** Quality assessment of the included studies using the AQUA tool. “Low,” “Unclear,” and “High” refer to risk-of-bias judgments for individual AQUA domains, whereas “Medium” and “High” in the final column refer to the overall methodological quality rating.

Study	Domain 1: Objectives and Study Characteristics	Domain 2: Study Design	Domain 3: Methodology Characterization	Domain 4: Descriptive Anatomy	Domain 5: Reporting of Results	Overall Methodological Quality
Parsons (1916) [22]	Unclear	Low	Unclear	Low	Low	Medium
Pühringer (1920) [29]	Unclear	Low	Unclear	Low	Low	Medium
Santos (1927) [17]	Low	Low	Low	Low	Unclear	Medium
Ahlborg (1931) [30]	Unclear	Low	Unclear	Low	Low	Medium
Skarby (1936) [31]	Low	Low	Unclear	Low	Low	Medium
Olivier (1951) [32]	Unclear	Low	Unclear	Low	Low	Medium
Papadatos (1980) [6]	Unclear	Low	Low	Low	Low	Medium
Sommerlad (1981) [23]	Unclear	Low	Unclear	Low	Low	Medium
Chung (1995) [24]	Low	Low	Low	Low	Low	High
Jelev (2007) [1]	Low	Low	Low	Low	Low	High
Natsis (2016) [25]	Low	Low	Low	Low	Low	High
Nathe (2011) [3]	Low	Low	Low	Low	Low	High
Chanda (2014) [33]	Unclear	Low	Unclear	Low	Low	Medium
Khadanovich (2025) [34]	Low	Low	Low	Low	Low	High
Present study	Low	Low	Low	Low	Low	High

AQUA domains are reported as risk-of-bias judgments (low, unclear, or high risk of bias), whereas the final column summarizes the overall methodological quality of each study.

**Table 3:** Morphometric characteristics of transclavicular canals. Values are reported as extracted from the original studies where available. For Santos (1927) [17], approximate mean ± SD values were derived from published range summaries under an assumption of approximate normality. Positional variables may refer either to the closest canal margin, the canal center, or the nearest canal orifice, depending on the source study; derived ratios are indicated in footnotes. Summary values are canal-count-weighted descriptive means rather than formal meta-analytic pooled estimates because the source studies reported sparse morphometric data with incompletely identifiable pairing.

Study	Number of Canals	Medial Distance (Mean ± SD)	Lateral Distance (Mean ± SD)	Lateral Distance/Total Clavicle Length	Canal Length (Mean ± SD)	Canal Width (Mean ± SD) ⁋
Santos (1927) [17] ⁂	30	NR	61.0 ± 4.9	0.42 ± 0.04 ⁑	7.5 ± 2.2	NR *
Chanda (2014) [33]	1	84	65	0.436 †	NR	NR
Khadanovich (2025) [34]	22	68.6 ± 10.6	60.4 ± 17.6	0.468 ± 0.082	4.9 ± 1.6	2.0 ± 0.6
Present study	3	73.4 ± 9.5	60.0 ± 5.2	0.441 ± 0.012	5.9 ± 2.4	1.5 ± 0.8
Descriptive summary of available data	56	69.7 ± 10.6	60.8 ± 11.5	0.440 ± 0.063 ‡	6.4 ± 2.3	1.94 ± 0.63

NR: not reported. † Derived as lateral distance/(medial distance + lateral distance) from the reported mean distances. ‡ Canal-count-weighted descriptive summaries only; see Methods for derivation details. ⁋ Khadanovich et al. [34] measured canal width digitally on calibrated photographs, whereas the present-study value represents aggregate transverse wire span, an operational lower-bound estimate of the wider internal lumen dimension. Because the methods are related but non-equivalent, their combined value is an exploratory descriptive summary only. ⁂ Santos-derived estimate reconstructed from published summaries; see Methods [17]. ⁑ Approximate acromial ratio derived from Santos’ grouped clavicle-length and orifice-distance summaries; see Methods [17]. * Santos reports orifice diameters [17], which were not entered into the modern canal-width column.

## Data Availability

The original contributions presented in this study are included in the article and Supplementary Material. Further inquiries can be directed to the corresponding author.

## Supplementary Materials

The following supporting information can be downloaded at: https://www.mdpi.com/article/10.3390/jfmk11030289/s1, Figure S1: Representative caliper measurement of the external dimensions of a supraclavicular foramen in a dry clavicle. The opposing caliper jaws were positioned against the external margins of the opening at its widest visible dimension. This caliper-based measurement characterizes the external opening and is distinct from the wire-defined measurement of internal canal patency; Figure S2: Galbraith plot for the per-clavicle prevalence meta-analysis. Each point represents one study, with study precision $\left(1/SE\right)$ shown on the abscissa and the standardized Freeman–Tukey transformed prevalence shown on the ordinate. The fitted regression line represents the overall relationship between standardized effect and precision, and the shaded region represents the corresponding 95% confidence limits. Studies falling outside or close to the limits may contribute disproportionately to between-study heterogeneity. Some study-level deviations were observed; however, the Galbraith plot is not specific evidence of publication bias and was interpreted together with the remaining, partly discordant asymmetry diagnostics; Figure S3: Funnel plot with trim-and-fill sensitivity analysis for the per-clavicle prevalence meta-analysis. The abscissa shows Freeman–Tukey transformed prevalence, and the ordinate shows the corresponding standard error, with more precise studies appearing toward the top of the plot. The diagonal lines represent the pseudo-95% confidence limits, and the vertical line indicates the random-effects pooled estimate. Although the observed studies show some visual asymmetry, the trim-and-fill procedure did not impute any additional studies and did not modify the pooled estimate. This analysis was interpreted as a supplementary diagnostic because funnel-plot geometry and trim-and-fill results may be influenced by between-study heterogeneity and the limited number of included datasets; Figure S4: Study-level estimates of maximum reporting bias in the per-clavicle prevalence synthesis. Squares represent the study-specific model-derived estimates, horizontal lines represent their 95% confidence intervals, and the diamond represents the random-effects pooled estimate. The pooled maximum reporting-bias estimate was 6% (95% confidence interval, 2–15%), with substantial between-study heterogeneity (I^2^ = 100%). This analysis represents a model-based upper-bound assessment of possible selective reporting and should not be interpreted as a direct estimate of the number or effect of unpublished studies; Figure S5: Representative dry clavicle showing a true supraclavicular canal and separate nutrient foramina. The supraclavicular canal is seen superiorly, with two external openings connected by a complete transosseous passage. Inferiorly, two smaller nutrient foramina are visible; unlike the supraclavicular canal, these are blind-ending vascular openings and do not form a complete traversing canal. The image illustrates the morphological distinction between supraclavicular canals and ordinary nutrient foramina. Scale in centimeters; Table S1: Full-text studies excluded after eligibility assessment, with reasons for exclusion; Table S2: Individual morphometric and wire-based characteristics of the three complete transclavicular canals. Linear dimensions are expressed in millimeters; projected obliquity is expressed in degrees.

## Abbreviations

The following abbreviation is used in this manuscript:

SCF	Supraclavicular foramen
