# Supplementary material for: Supraclavicular Foramina in Dry Clavicles: An Observational Study and Meta-Analysis of Prevalence and Laterality
Source: J Funct Morphol Kinesiol. 2026 Jul 24;11(3):289. doi: 10.3390/jfmk11030289 (PMC13510322; doi:10.3390/jfmk11030289)

Supplementary Material

Supplementary Table S1. Full-text studies excluded after eligibility assessment, with reasons for exclusion.

First author, year	Country	Study type / material	Why it appeared potentially eligible	Reason for exclusion from quantitative synthesis	Retained for discussion?
Jupiter, 2007	USA	Two-patient clinical case report	Title concerned supraclavicular nerve entrapment related to the clavicle	Excluded because the paper described post-traumatic entrapment due to clavicular fracture callus, not a congenital supraclavicular foramen / transclavicular canal series	Yes, as clinical support for secondary supraclavicular nerve entrapment
Sowmiya, 2016	India	Osteological study of 110 dry clavicles	Title referred to "neurovascular foramina" of the clavicle, raising possible relevance to supraclavicular nerve-related foramina	Study concerned ordinary clavicular nutrient foramina, not supraclavicular foramina/transclavicular canals; reported a common vascular foramen pattern rather than the rare SCF endpoint prespecified for this review	Yes, as a nomenclature caution
Viciano, 2017	Italy	Paleopathological case report of a single clavicle	Described congenital clavicular foramina/canal that could be confused with transclavicular canal	Excluded because the reported congenital clavicular canal/foramina likely reflected vascular inclusion during ossification rather than a supraclavicular nerve canal	Yes, as a differential-anomaly caution
Lorsuwannarat, 2023	Thailand	Cadaveric anatomical study; 64 shoulders from 32 adult cadavers	Anatomical study of supraclavicular nerve–clavicle relationships; reports nerve-course topography and clavicular safe zones	Did not explicitly report prevalence, morphology, or topography of supraclavicular foramina/transclavicular canals as prespecified review endpoints; focused on supraclavicular nerve branching and safe **zones**, with terminal branches described as passing over the clavicle	Yes

Supplementary Table S2. Individual morphometric and wire-based characteristics of the three complete transclavicular canals. Linear dimensions are expressed in millimeters; projected obliquity is expressed in degrees.

Panel A. External-opening morphology and position

Specimen	Side	Opening A				Opening B			
		Maximum opening length	Perpendicular opening height	Distance from acromial end	Distance from sternal end	Maximum opening length	Perpendicular opening height	Distance from acromial end	Distance from sternal end
1	Right	3.84	0.82	63.61	74.96	2.20	0.95	60.77	79.23
2	Left	3.09	0.89	64.87	79.56	3.00	0.97	62.64	81.55
3	Left	2.92	1.15	53.69	62.07	3.96	1.00	54.40	63.26

Panel B. Canal-level morphology and wire-based patency

Specimen	Side	Clavicle length	Largest-single-wire patency	Maximum simultaneous wire count	Aggregate transverse wire span	Acromial midpoint distance	Sternal midpoint distance	Acromial midpoint ratio	Canal length	Projected obliquity (degrees)
1	Right	144.45	0.2	2	0.4	62.19	77.10	0.431	8.55	30.0
2	Left	145.26	0.6	4	2.4	63.76	80.56	0.439	4.15	28.7
3	Left	118.80	0.4	4	1.6	54.05	62.67	0.455	4.86	14.2

Opening A denotes the opening identified as closer to the sternal end according to the sternal-end measurement; Opening B denotes the other opening. These labels describe position and do not imply the direction of neural passage.

Opening dimensions were measured as maximum length and perpendicular height.

Largest single-wire patency was defined as the diameter of the largest individual wire that traversed the complete canal. Maximum simultaneous wire count denotes the number of equal-gauge wires passed simultaneously in a side-by-side arrangement.

Aggregate transverse wire span was calculated as the largest single-wire diameter multiplied by the maximum simultaneous wire count. It represents an operational lower-bound estimate of the wider internal lumen dimension rather than a directly measured anatomical diameter.

Canal midpoint distances were calculated as the mean of the corresponding measurements for Openings A and B. The acromial midpoint ratio was calculated as the acromial midpoint distance divided by total clavicular length.

Supplementary Figure S1. Representative caliper measurement of the external dimensions of a supraclavicular foramen in a dry clavicle**.** The opposing caliper jaws were positioned against the external margins of the opening at its widest visible dimension. This caliper-based measurement characterizes the external opening and is distinct from the wire-defined measurement of internal canal patency.

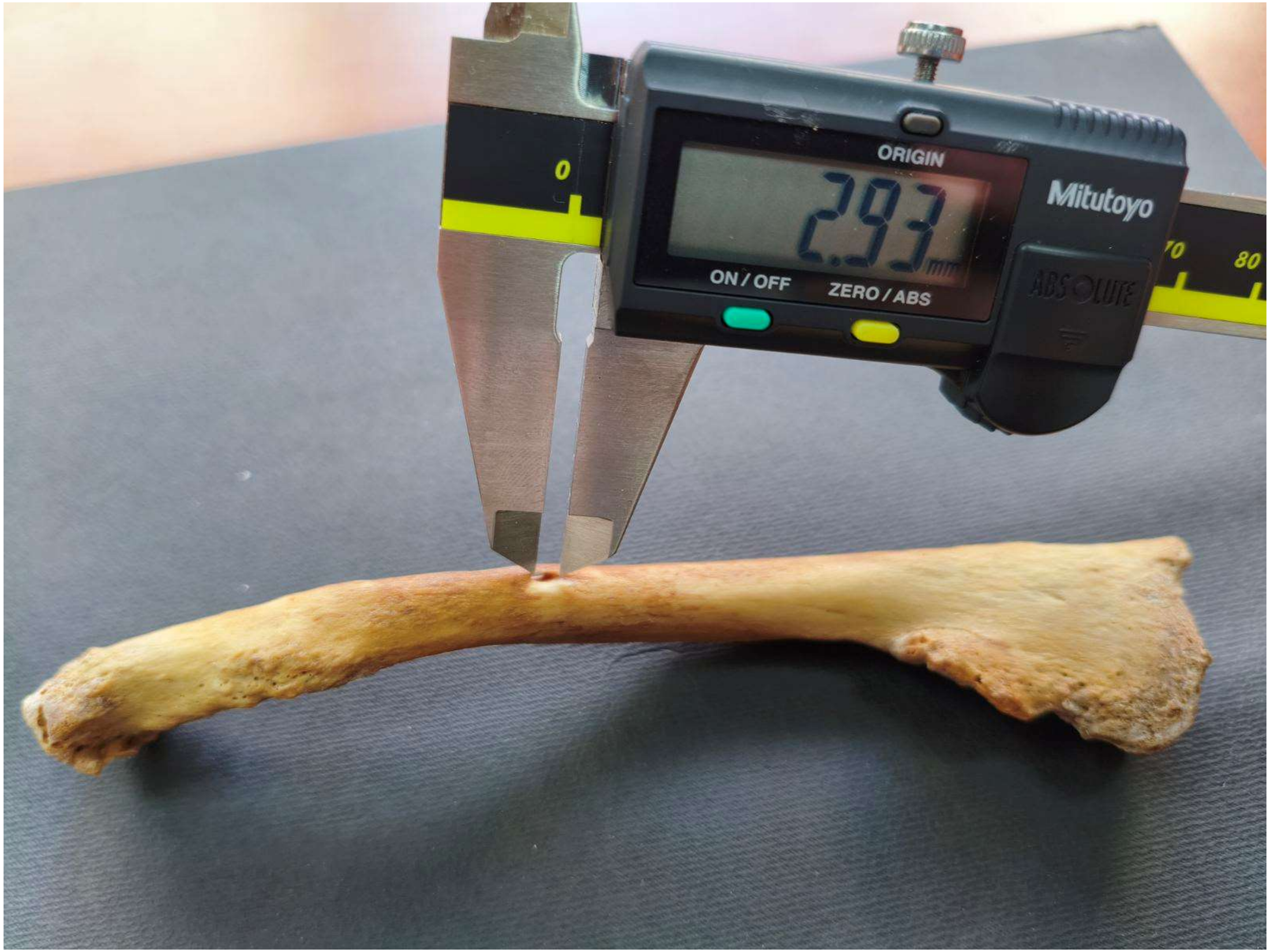

Supplementary Figure S2. Galbraith plot for the per-clavicle prevalence meta-analysis. Each point represents one study, with study precision ($1/SE$) shown on the abscissa and the standardized Freeman–Tukey transformed prevalence shown on the ordinate. The fitted regression line represents the overall relationship between standardized effect and precision, and the shaded region represents the corresponding 95% confidence limits. Studies falling outside or close to the limits may contribute disproportionately to between-study heterogeneity. Some study-level deviations were observed; however, the Galbraith plot is not specific evidence of publication bias and was interpreted together with the remaining, partly discordant asymmetry diagnostics.

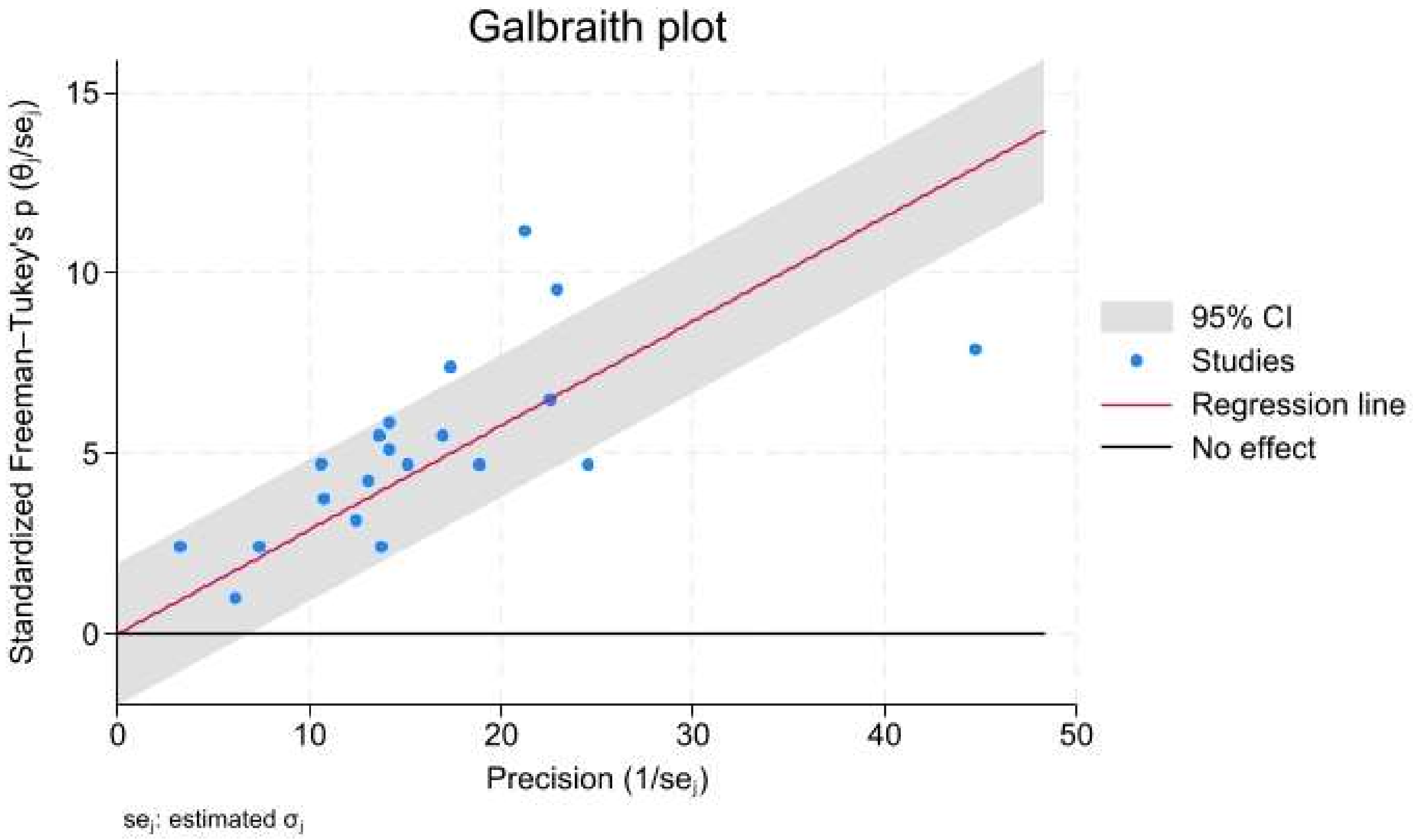

Supplementary Figure S3. **Funnel plot with trim-and-fill sensitivity analysis for the per-clavicle prevalence meta-analysis.** The abscissa shows Freeman–Tukey transformed prevalence, and the ordinate shows the corresponding standard error, with more precise studies appearing toward the top of the plot. The diagonal lines represent the pseudo-95% confidence limits, and the vertical line indicates the random-effects pooled estimate. Although the observed studies show some visual asymmetry, the trim-and-fill procedure did not impute any additional studies and did not modify the pooled estimate. This analysis was interpreted as a supplementary diagnostic because funnel-plot geometry and trim-and-fill results may be influenced by between-study heterogeneity and the limited number of included datasets.

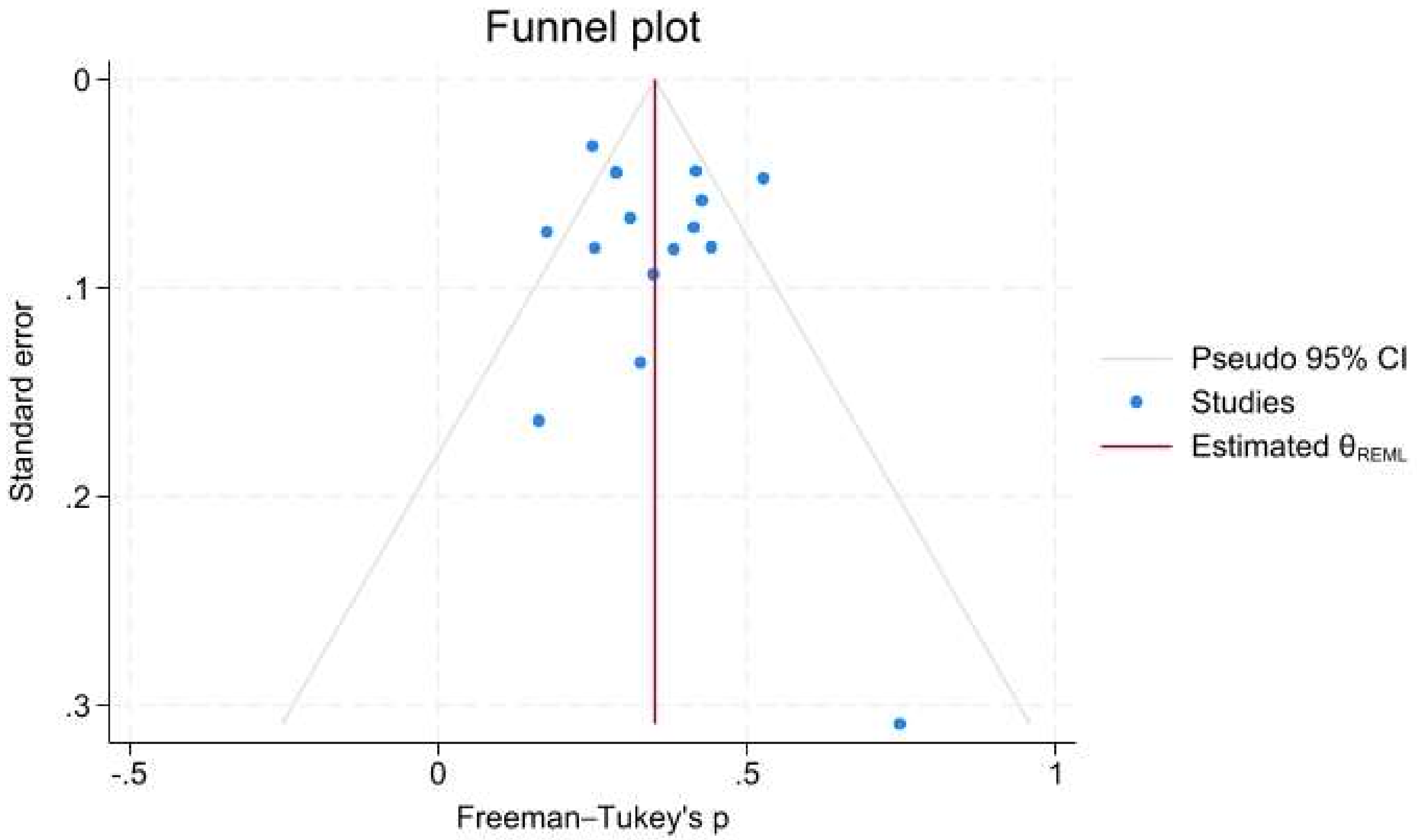

Supplementary Figure S4. **Study-level estimates of maximum reporting bias in the per-clavicle prevalence synthesis.** Squares represent the study-specific model-derived estimates, horizontal lines represent their 95% confidence intervals, and the diamond represents the random-effects pooled estimate. The pooled maximum reporting-bias estimate was 6% (95% confidence interval, 2–15%), with substantial between-study heterogeneity ($I^2 = 100\%$). This analysis represents a model-based upper-bound assessment of possible selective reporting and should not be interpreted as a direct estimate of the number or effect of unpublished studies.

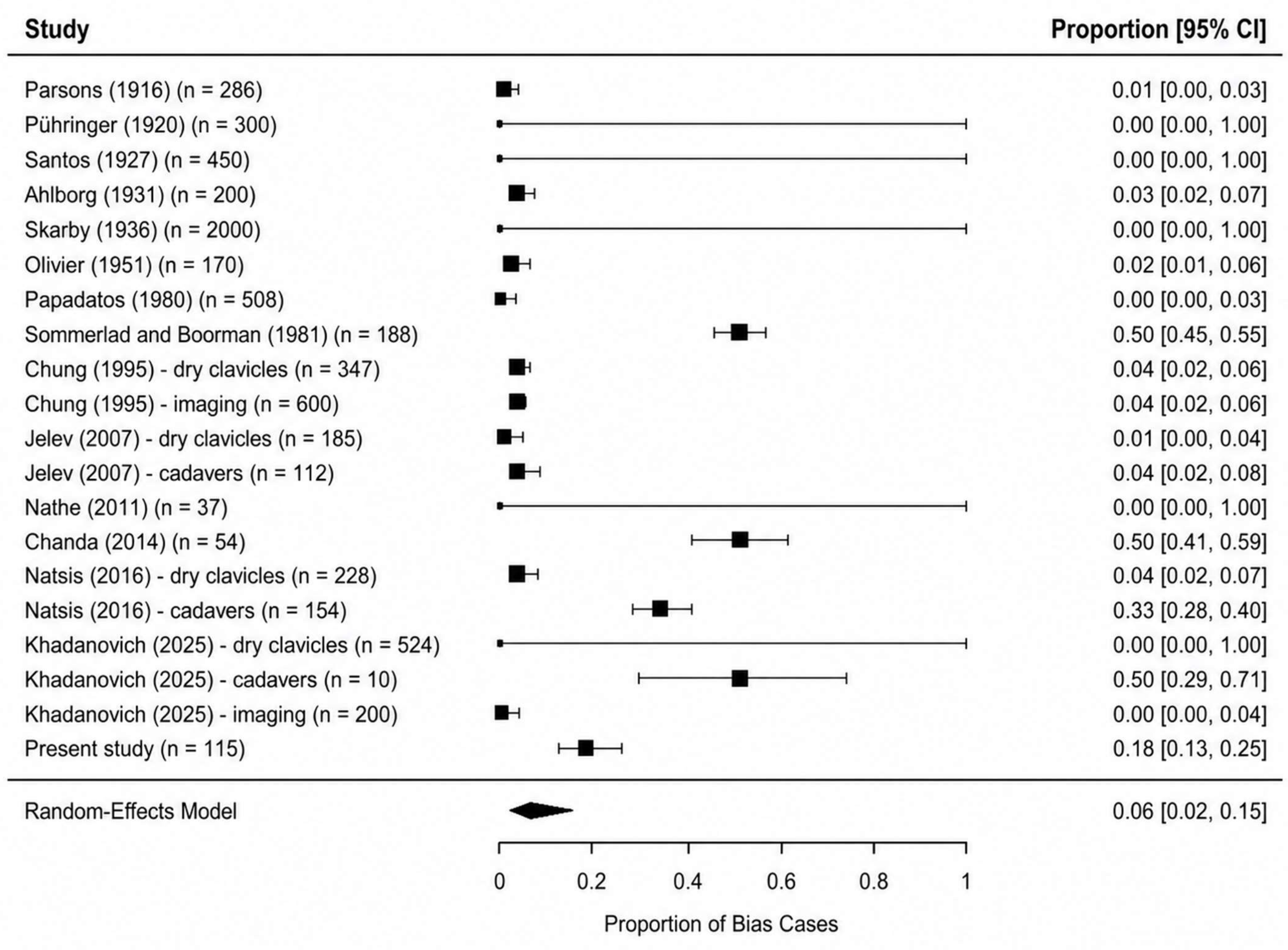

Supplementary Figure S5. Representative dry clavicle showing a true supraclavicular canal and separate nutrient foramina. The supraclavicular canal is seen superiorly, with two external openings connected by a complete transosseous passage. Inferiorly, two smaller nutrient foramina are visible; unlike the supraclavicular canal, these are blind-ending vascular openings and do not form a complete traversing canal. The image illustrates the morphological distinction between supraclavicular canals and ordinary nutrient foramina. Scale in centimeters.

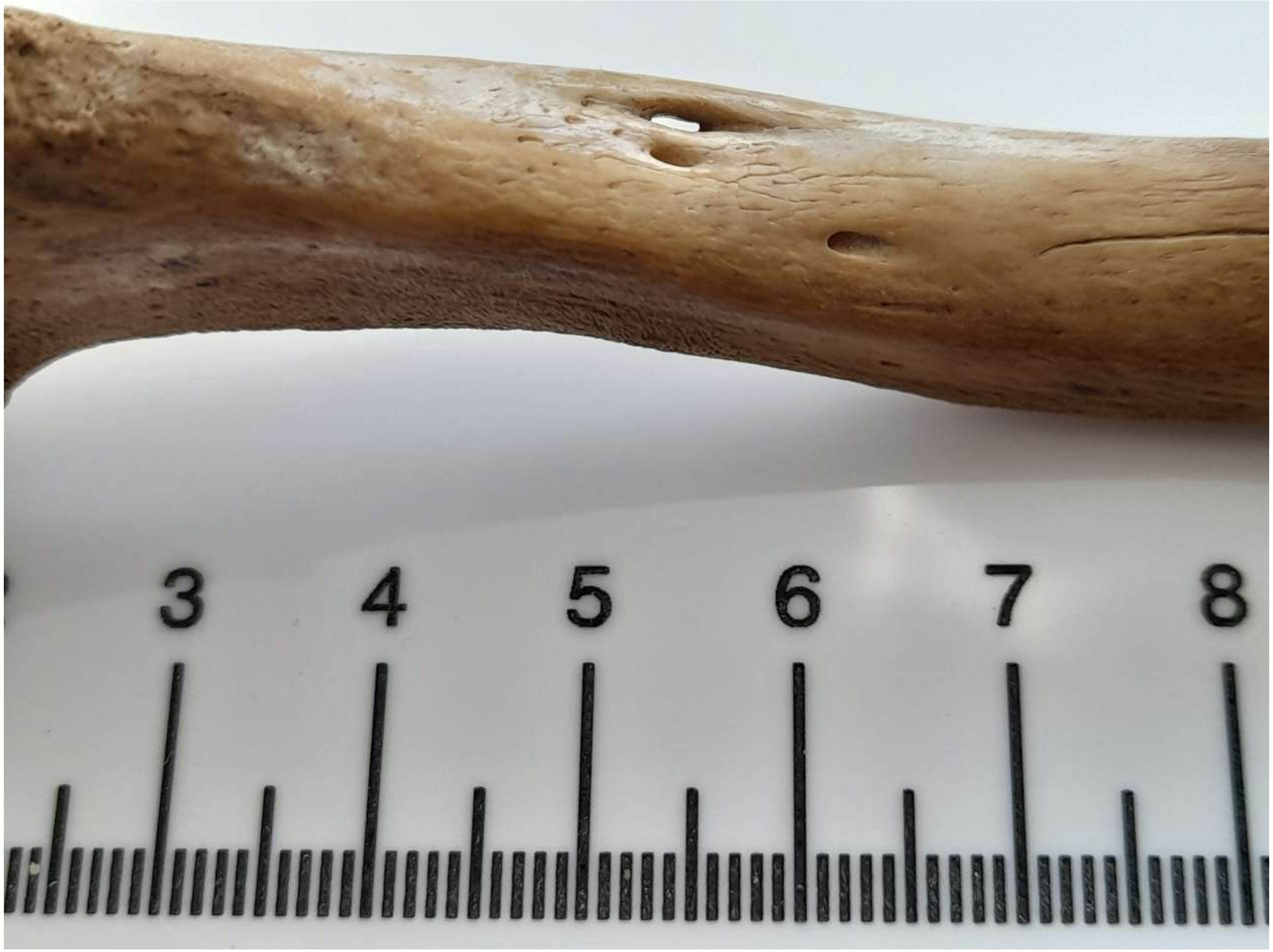

Supplement: Supplementary file 1 [file jfmk-11-00289-s001.zip › jfmk-4439298-supplementary.pdf]
